# A randomized controlled trial to examine the effect of two teaching methods on preschool children’s language and communication, executive functions, socioemotional comprehension, and early math skills

**DOI:** 10.1186/s40359-019-0325-9

**Published:** 2019-09-05

**Authors:** Tove Gerholm, Petter Kallioinen, Signe Tonér, Sofia Frankenberg, Susanne Kjällander, Anna Palmer, Hillevi Lenz-Taguchi

**Affiliations:** 1 Dept of Linguistics, Stockholm University, Stockholm, Sweden; 2 Dept of Child and Youth Studies, Stockholm University, Stockholm, Sweden

**Keywords:** Intervention, Preschool, Language skills, Communication skills, Executive functions, Auditory selective attention, Socioemotional comprehension, Early math skills, Group-based learning, Digital learning

## Abstract

**Background:**

During the preschool years, children’s development of skills like language and communication, executive functions, and socioemotional comprehension undergo dramatic development. Still, our knowledge of how these skills are enhanced is limited. The preschool contexts constitute a well-suited arena for investigating these skills and hold the potential for giving children an equal opportunity preparing for the school years to come. The present study compared two pedagogical methods in the Swedish preschool context as to their effect on language and communication, executive functions, socioemotional comprehension, and early math. The study targeted children in the age span four-to-six-year-old, with an additional focus on these children’s backgrounds in terms of socioeconomic status, age, gender, number of languages, time spent at preschool, and preschool start. An additional goal of the study was to add to prior research by aiming at disentangling the relationship between the investigated variables.

**Method:**

The study constitutes a randomized controlled trial including 18 preschools and 29 preschool units, with a total of 431 children, and 98 teachers. The interventions lasted for 6 weeks, preceded by pre-testing and followed by post-testing of the children. Randomization was conducted on the level of preschool unit, to either of the two interventions or to control. The interventions consisted of a socioemotional and material learning paradigm (SEMLA) and a digitally implemented attention and math training paradigm (DIL). The preschools were further evaluated with ECERS-3. The main analysis was a series of univariate mixed regression models, where the nested structure of individuals, preschool units and preschools were modeled using random variables.

**Results:**

The result of the intervention shows that neither of the two intervention paradigms had measurable effects on the targeted skills. However, there were results as to the follow-up questions, such as executive functions predicting all other variables (language and communication, socioemotional comprehension, and math). Background variables were related to each other in patterns congruent with earlier findings, such as socioeconomic status predicting outcome measures across the board. The results are discussed in relation to intervention fidelity, length of intervention, preschool quality, and the impact of background variables on children’s developmental trajectories and life prospects.

**Electronic supplementary material:**

The online version of this article (10.1186/s40359-019-0325-9) contains supplementary material, which is available to authorized users.

## Background

A comprehensive preschool system has the unique possibility to enhance social, emotional and cognitive skills, as well as fostering general behaviors deemed important by society, such as participative, democratic citizenship. Preschools are not available worldwide and where they exist, differences can be great in a number of ways, such as whether they are subsidized or not. In countries like Sweden, where 84% of the one- to three-year-old children and 95% of the four- and five-year-olds [1] are enrolled in whole-day preschool services, the system reaches close to all children, regardless of socioeconomic status (SES), languages or family situation, during years essential for learning. In order for preschools to enhance children’s abilities and skills, the educational services provided need to be of a “good enough” quality in terms of teacher/child ratio, educated staff, meaningful activities including time for play, positive interactions between children and adults, access to inspiring learning materials and environments, etc. [2].

For a long time, intervention studies have been the main way to investigate the use and effectiveness of early education internationally [3, 4]. The skills most often targeted, since they have proven essential for later outcomes in children and adolescents [5, 6], are executive functions (including auditory selective attention, [4]), socioemotional skills, language and literacy, as well as math [7–11]. Evidence from intervention studies from different parts of the world indicate that all of these skills, together with IQ and self-regulation, can be enhanced through pedagogical training [12–14]. In an RCT study of 759 preschool children, Blair and Raver [13] concluded that not only did the intervention have an effect on the targeted ability self-regulation, but the children also improved in mathematics, reading and vocabulary with results increasing into first grade. Neville et al. [4] found significant effects in an ERP-paradigm of auditory selective attention in a sample of 33 Head Start children following 8 weeks of intervention. In an RCT study also targeting Head Start children, Nix et al. [15] showed that socioemotional skills could be enhanced through a REDI (Research-Based, Developmentally-Informed) enrichment intervention. A couple of studies have also been able to demonstrate effects from preschool self-regulation training that lasted well into adulthood [16, 17].

In Sweden and the Scandinavian countries, intervention research performed with children prior to compulsory school is less common. This is an important observation, as the different circumstances for preschool services worldwide make comparisons between intervention studies potentially skewed. Nemmi et al. [18] showed in a sample of 55 six-year-olds that grit predicts significant improvements in working memory, as a result of an eight-week training program including working memory and early math tasks. Thorell et al. [19] investigated working memory and inhibition in a sample of 65 Swedish preschool children aged four to five, using an intervention with 5 weeks of either visuo-spatial training or inhibition training for 15 min a day using computer games. The results showed significant improvement in working memory as well as transfer effects on attention for these children, whereas inhibition training did not yield results. There was no follow-up to check for long-term effects in this sample, however, Klingberg et al. [20] could show effects at least 3 months after a completed study on school-aged children’s working memory. In Denmark, a country that is similar to Sweden in many ways, in particular as it comes to preschool attendance and a general focus on socialization and play in the preschool curriculum, Bleses et al. [21] enrolled 5,436 children aged three to six in an RCT study targeting pre-literacy skills and language and found significant results for pre-literacy skills, albeit not for language, after a 20-week intervention.

This said, many studies, both internationally and in the local Scandinavian context, also come to diverging results when investigating the same or similar skills [22, 23]. Long-term effects of intervention studies have also been hard to find [24, 25]. However, adding children’s backgrounds as a variable resolve some of the divergences and accounting for preschool quality could help explain yet others.

Starting with child background, the evidence has long been piling up that socioeconomic status plays a key role in how a child will develop through the preschool years and beyond [26, 27]. For example, Blair and Raver [13], who found effects on self-regulation, literacy, mathematics and science learning through using the educational approach Tools of the Mind [28], could also conclude that the effect was most prominent in the group of children starting out in low-SES environments. Similar findings stem from Neville et al. [4] who, in their intervention study using ERP-responses and targeting Head Start schools, found a significant increase in the children’s results on auditory selective attention. Other intervention studies have come to the same conclusions on executive functions and academic abilities [5, 6, 12, 29–31]. Further, intervention studies performed in preschools including high-SES children as well, have not been able to replicate the findings [32].

Socioeconomic background is a complex concept, which calls for some caution in interpreting intervention results. Whereas most interventions appear to have a larger effect on children from low-SES backgrounds, there is also evidence pointing the other way. When targeting specific skills like language and literacy, low-SES children benefited less than their more fortunate peers from interventions in studies by Buysse et al. [33] and Marulis and Neuman [34]. Adding to the confusion, a meta-analysis of the National Early Literacy Panel [35] reported the opposite results on pre-literacy, as low-SES children showed larger outcome effects than high-SES children. Bleses et al. [7] suggest an interpretation where these mixed results could depend on different groups of children needing different forms of interventions, such as a higher intensity for children with particular risk factors. One potential cause of differing results is also the way SES is measured. While some studies use income and education, others use only income or educational level, yet others base their classification on living area (e.g., wealthy/poor neighborhood), and so on. To further clarify how different studies reach different conclusions when investigating the same or similar phenomena, transparency of how the different concepts – like SES – is measured, together with clear description of the implementations provided and, in particular, the fidelity of the implementation, need be addressed.

Turning to the other main explanatory factor of diverging results, we find that adding high quality Early Childhood Education and Care provisions (henceforth ECEC) as a variable makes long-term effects of preschool curricula more conclusive [36]. An example is a longitudinal study of 141 preschool provisions in the U.K. investigating the effects of preschool quality (measured with the environmental ECERS scale; [37]) on eleven-year-olds. Sylva et al. [38] showed that preschool quality significantly predicted most measured outcomes when considering key child and family variables. Children who had attended low quality preschools, however, did not significantly differ on cognitive and behavioral scores from children with no preschool experiences at all. At the same time, findings from a Norwegian study indicate that simply attending preschool for long enough period of time could be essential. Havnes and Mogstad [39] analyzed data from a ‘natural experiment’ in Norway based on a preschool reform of subsidized child care, comparing the long-term effects on children in municipalities who extensively expanded their preschool provisions with those who did not decide to do so. The results showed that preschool attendance had strong positive effects on educational attainment, labor market participation and reduced dependence on welfare. As there is no information as to the quality of the Norwegian preschools, the different conclusions are hard to conjoin.

As a part of the Norwegian Agder project, Rege et al. [40] investigated preschool quality, focusing on the structural quality of the services; i.e., child-teacher ratio, center size and the tenure of the director, when evaluating school readiness in 627 five-year-olds enrolled at 67 ECEC centers across Norway. Although the differences in quality cannot be ruled out as effects of unobservable background variables, the study demonstrates significant differences in school readiness skills in five-year-olds. Since this study only measures structural quality, the authors conclude that the results must be interpreted with caution. In a Danish study [41] aiming to investigate the effects of preschool quality (measured through class size, child-staff ratios, and teacher education), 30,444 children who had attended a formal preschool institution had their grades from ninth grade correlated to their earlier preschools’ qualities. Findings suggest that an increase in structural conditions only have modest effects on children’s development in general. However, on specific scales, significant findings emerged, such as boys benefitting more than girls from formal teacher training.

Albeit from similar settings and cultures, the Scandinavian studies end up with some inconsistent results. Bauchmüller and colleagues’ [41] results of modest but persistent associations between quality of preschool services and outcomes by the end of ninth grade of schooling, contrasts Chetty et al. [42], who found that effects of preschool quality on cognitive skills will fade before the children reach their teens. A Danish study by Gupta and Simonsen [43] on non-cognitive outcomes of preschool vis-à-vis home care, had results showing that boys whose mothers had a low educational level benefited more than girls from an intervention (see also [41]). However, Havnes and Mogstad [39] also found that girls benefitted more in the long run than boys in terms of education attainment and labor market participation and had a lower level of social welfare. It is currently not clear why there are such immense differences in results from different intervention studies. Even in studies targeting the same ages and in the same or a similar cultural setting, specific skills appear to be enhanced in some studies but not in others. The array of explanatory factors suggested in earlier research and cited above are: children’s socioeconomic background, children’s sex and age, fidelity of intervention and implementation of intervention, number of hours in preschool, quality of preschool (as measured by e.g. ECERS), scripted vs non-scripted instructions, and assessment of targeted skills.

The present study set out to investigate the effectiveness of two pedagogical methodologies, which to some degree were already in use within the Swedish preschool context, though they had not yet been scientifically evaluated. One is based on socioemotional learning [44, 45], mainly group-based and with a focus on interaction, whereas the other is more individual as children work with digital tablets to enhance particular skills and/or learn to control and understand their bodies [4, 10, 46]. Both methodologies are believed to enhance children’s language and communication, EF, socioemotional comprehension and math, albeit to different degrees and in different ways, and they are both advocated by the National Agency for Education by way of the preschool curriculum [47]. Nevertheless, they are often described as in conflict within the Swedish preschool setting. By performing an RCT intervention, comparing these methodologies in a boosted version to a control group where presumably a mixture of methodologies is in use, the present study aimed to deepen our understanding of how particular skills are enhanced in preschoolers. Following Neville et al. [4] whose research highlight two themes central to us: SES and executive functions, we included an ERP test of auditory selective attention as a complement to the behavioral test battery. By including SES, age, sex, number of hours at preschool and quality of preschool among the variables, and by carefully monitoring fidelity of implementation and assessment, we further hoped to be able to add to prior research by clarifying the relation between background factors and preschool outcome.

## The aims, interventions, questions and hypotheses of the study

### Aims

The present study aimed to investigate which – if either – of two intervention pedagogical methods would prove most suitable to enhance children’s language and communication, executive functions, socioemotional comprehension, and early math skills in preschool settings. The full details of the study set-up and implementation are described in a Study Protocol [48]; however, for the convenience of the reader the main parts of the study will also be covered in the following paragraphs. The sample was unselected within the enrolled preschools, including all children who opted in for participation regardless of potential difficulties or developmental disorders. The study was performed in 29 preschool units involving all in all 431 children and 98 educators, in a municipality outside Stockholm, Sweden. The objective was to compare a group-based socioemotional learning strategy, henceforth referred to as SEMLA (socioemotional and material learning, [45]) with an individual digital learning paradigm called Digital Individual Learning for body-and-mind (DIL).

### Interventions

The SEMLA intervention was designed to enhance children’s language and communication, EF, socioemotional comprehension, and early math skills as part of an investigative learning strategy with emphasis on the STEAM subjects (Science, Technology, Engineering, Art and Mathematics, [49]), specifically focusing on early mathematics. This was done as part of a group-based collaboration designed to explore the overarching problem of how humans might live and get around 100 years from now, using a manifold of construction materials, digital tools, documentation and meta-reflecting practices [50]. In practice, SEMLA addresses socioemotional comprehension through face-to-face interaction [44], as well as in the creative handling of various forms of materials and artefacts used as multimodal tools for exploration and construction [51–53]. The emotional engagement in learning [54] was emphasized and used as an important driving force as the children engaged in hands-on investigations involving diverse materials and artefacts. This driving force would, in itself, create a positive learning ground, engaging children and help motivate them for learning [54]. As a group-based strategy, SEMLA is believed to enhance language and socioemotional comprehension by having the children listening to each other, expanding and reflecting on other’s utterances of verbal as well as nonverbal matters [55, 56]. New words and/or concepts were introduced by the teachers and elaborated on in relation to both the overarching problem and the more specific problems emerging in the process of constructing and investigating [50]. Executive functions, including auditory selective attention were believed to be enhanced through these processes of verbally mediated reflection and focused attention – on materials, exploration themes, difficulties encountered, translations between words, meanings and materials – in combination with the close scaffolding from the educators [57–59].^1^ The overarching problem of investigating how we might live and get around 100 years from now was introduced to smaller groups of six to eight children at a time, and targeted early math, as it contained instances of measuring, estimations, distances, and engineering and constructions of vehicles and buildings, thought to be part of a future life [49].

The second intervention, DIL, focused on individual training intended to enhance children’s executive functions, including auditory selective attention and self-regulation, and early math skills [60, 61]. More specifically, the intervention was developed based on the theoretical understanding of self-regulation and early math as developing interdependently [10, 62]. DIL had two components: an adaptive, interactive math game and a set of attention-enhancing body-and-mind activities.

The interactive math game, *The Magical Garden* (MG, [46])^2^ was played on digital tablets with headphones. It focuses on early math and number sense and is administered online by the Education Technology Group at Lund University [46]. The main theme of the game is for the child to solve math problems in order to collect water to create a flourishing garden. The game includes a teachable agent (TA) based on a learning-by-teaching methodology. The child is encouraged to teach the TA early math. The game design and narrative are adaptive, and the game progressively advances in difficulty, with feedback provided to motivate the child [57]. The game has been investigated scientifically, focusing on functionality, such as the TA, scaffolding, gaming strategies, eye movement and inhibition [62, 64]. The two tasks in combination were believed to improve self-regulation as well as early math skills [10, 65].

The body-and-mind exercises (Brain Development Lab,^3^ cf. [4]) were introduced by the educators and included a package of 12 activities focused on self-regulation. Specifically, they targeted attention, executive functions and meta-reflection by means of strategically designed metaphors [67] that corresponded to the design of the MG. The exercises were inspired by the child component of the evidence-based program Parents and Children Making Connections - Highlighting Attention [4]. The activities aimed at teaching children strategies for handling and controlling their bodies and minds and focused on training attention, breath control, avoiding distractions and improving body control, as well as on metacognition. For example, “The Bird Breath” poster features a metaphor designed with the same characters as in the MG and teaches children to take a deep breath to regain focused attention.^4^ The activities were introduced so as to gradually enhance the level of difficulty. The teacher scaffolds each child at his/her level throughout the activity.

The two interventions were compared to a control group in preschools where the daily pedagogical work was carried out as usual. The staff in the control group filled out a self-evaluative tool-kit, BRUK [68], administered by the Swedish National Agency for Education [69], which was aimed at enhancing motivation in the staff randomized to the control group.

### Research questions

The study set out to answer the following questions: 1) What are the effects of the two different pedagogical methods (SEMLA and DIL) on language and communication, executive functions, socioemotional comprehension, and early math skills? 2) How do any observed effects in these areas differ between the two interventions? 3) To what extent are any observed effects mediated by language and/or EF? 4) To what extent are any observed effects moderated by background variables like sex, age, preschool start etc.? 5) To what extent are the background variables related to the outcome variables? 6) To what extent are the outcome variables related to each other? 7) Do any observed effects of the interventions differ in terms of strength and variation?

### Hypotheses^5^

Our general hypothesis for the project was that both SEMLA and DIL would have a greater impact on the children’s development of language, communication, EF, math and socioemotional comprehension than would the practice as usual in the control groups. However, the difference between the interventions made us hypothesize that DIL would have a stronger effect on math (due to the specific training of math through the digital app), whereas SEMLA would have a stronger effect on language, communication and socioemotional comprehension due to these abilities being at the forefront of the SEMLA approach. As all of the preschools were evaluated with the ECERS-3, our assumption was that preschools scoring high for quality would also get a better result with the implementations in all areas tested.

Background factors come together in particular patterns e.g. [70, 71]. Following prior research, our hypotheses in regard to this was that age would be correlated to language level (as measured by SCDI; [72]). High SES would, in a similar manner be correlated to SCDI scores, since earlier research has found a connection between middle-class parents and children’s higher language proficiency. High SES was further expected to yield higher scores on EF and language at pre-testing. Other language-related findings made us expect that children with Swedish as their strongest language would have a higher SES than children with other L1 than Swedish. This is based on the assumption that these children might have arrived more recently in Sweden and be less established in terms of education and employment (see e.g. [73]). High-SES children (where both parents in the majority of cases have full-time employment) were also expected to have longer days at preschool, hopefully making them more affected by good pedagogical practices. Related to this, multilingual children were expected to enter preschool at a later age than Swedish monolingual children (in turn leading to multilingual children having less time to be influenced by pedagogical training in preschool). A trivial hypothesis was further that children with Swedish as their strongest language would have an easier time both partaking in and understanding the tasks where language was essential for performance. This was particularly the case for the math task. A high score on language tasks pre-intervention was also expected to correlate with a higher outcome score on socioemotional comprehension, as socioemotional comprehension is expressed most centrally through language [74–76].

Low SES was expected to have a moderating effect on language, EF, and socioemotional comprehension, since this is what earlier research has found [13, 35]. Guided by prior research, we also expected girls to perform better on EF, language, communication, and socioemotional comprehension than boys [44, 77–80]. As some research has found multilingualism to be positively correlated with EF [81, 82], we hypothesized that we would find the same relation.

Some variables were further expected to have a mediating effect, and based on prior research [83, 84], we expected EF to facilitate improvement in language, communication, math, and socioemotional comprehension regardless of intervention. Conversely, language and math were also expected to have a mediating effect on EF [10]. EF scores at pretesting were also hypothesized to have a moderating effect on any observed intervention effects with regard to EF in both SEMLA and DIL, so that a child with an initially low EF score would benefit more from the interventions in regard to EF than would a child who had already scored high in this domain at the start [4, 30].

## Methods

### Study design

The project was a three-armed, cluster-randomized, controlled study, implemented in three waves during a period of 10 months (September 2016 to June 2017), and was analyzed using mixed models regressions [85]. The protocol for this study was published in advance of its completion [48] and both the protocol and study are reported according to CONSORT guidelines [86]. The main research questions were initially tested as planned, using these univariate regressions (see Results). Because of problems with multicollinearity we also reformulated the analysis to a multivariate version where the composite measures of the planned analysis were entered as separate variables (see Results). However, the study also produced data suitable for qualitative analyses. The video recordings of the testing situations form the bases for transcriptional work through which we measured verbal and nonverbal language and communication skills among the children.

### Recruiting

A municipality that already had an ongoing cooperation with Stockholm University was asked to participate in the study. All 30 preschools run by the municipality were invited and 18 preschools opted in. In order for a preschool to be accepted, all involved preschool staff needed to sign a written consent form in which they stated their interest in participation and their understanding of the conditions of the randomization that would determine to which intervention or control they would be assigned.

Following information meetings at the different preschools, the guardians of 431 children (223 girls) signed up to let their children participate in the testing procedures of the project. Parents were not asked to evaluate or take a stand concerning the interventions as such, as these were regarded as part of a regular preschool curriculum. All participating parents had to fill in a background document for their child, including information such as family situation, family income and education, languages spoken in the family, time spent at preschool, number and age of siblings, medical history of the child, hereditary language-related conditions in the family, etc. The questionnaire was delivered in sealed envelopes to the parents and returned anonymized in prepaid envelopes directly to the university.

The 18 preschools consisted of 29 units in all, where a unit could include between seven and 30 children. This was a consequence of the project only targeting children from 4 years of age, as some units had mixed groups of three- and four-year-olds, meaning that the number of four-year-olds in some units could be very low. In order to participate in the study, a unit had to consist of at least seven children. In one case, there were only two four-year-olds in a unit, so that the preschool merged two units, resulting in a total of 28 participating units. Some preschools had many units while others had only one. The randomization was conducted at the unit level and took into account the number and size of units the preschool had. For example, a single preschool was not allowed to have both interventions, since the risk of contamination between interventions was deemed to be high if units were adjoined physically or if siblings/friends participated in different interventions. Thus, in a preschool with many units, these could be randomized to one of the interventions or to the control. Yet another condition for the randomization was to have as equal a distribution of ages as possible. For SEMLA, the age range was 49–74 months, for DIL 46–74 months and for the control, the age range was 44–74 months at pretesting.

One consequence of making the intervention in three waves was that randomization could not allow for all variables related to the children, since we did not have all information at the same time. One example is socioeconomic status, as we did not know during the first intervention period exactly which preschools or which children would be involved in wave two. During wave two we did know which preschools had signed up for the third wave, but we did not know which children would be involved, as parents were informed and accepted/declined participation in close proximity to the start of each intervention.^6^

### Sample

The units, interventions and background information on the children are presented in Table 1. The original sample consisted of 431 children (223 girls and 208 boys) with a mean age of 62 months. A majority of the children came from higher SES backgrounds. The sample was linguistically diverse, with 33% of the children having additional language(s) in the home environment and a total of 49 different languages being represented. English, Spanish, Arabic, Kurdish and Polish were the most frequent languages occurring in the children’s home environment apart from Swedish. A vast majority of children lived in two-parent households. Children had started preschool at 1;6 years on average and spent an average of 38 h/week at preschool. There were cases were caregivers did not answer all of the questions in the background questionnaires, thus there are missing data points for children’s age and SES (see also Table 1).

**Table 1:** The total number of participants were 431. Mean age was 62 months. The SEMLA group had a larger proportion of multilingual children than the other intervention groups. SES was generally high in the sample but differed significantly between intervention groups. A majority of children lived in two-parent households. Weekly preschool attendance was generally high and significantly higher in control than in SEMLA

	SEMLA	DIL	Control
Children, *n* = 431	137^*a*^	155^*a*^	139^*a*^
Child characteristics			
% boy, *n* = 431	54	47	46
Mean age in months (SD), *n* = 417	62 (6)	61 (7)	63 (7)
% multilingual, *n* = 431	53	27	22
Family characteristics			
SES, median, *n* = 393	7	8	9
% two-parent household, *n* = 431	89	88	91
Preschool attendance			
Mean age at preschool start (SD), *n* = 411	18 (9)	18 (6)	17 (5)
Mean preschool hours/week (SD), *n* = 370	37 (7)	37 (6)	39 (6)

*a*. Note: The uneven group sizes arose because preschool units have different sizes

The distribution of girls and boys did not differ significantly between groups (Kruskal-Wallis test, χ^2^ = 4.273, *p* = 0.12, df = 2), and there were no significant differences with regard to age at preschool start. However, despite random assignment, there were some significant differences between intervention groups. With regard to age, children in DIL were significantly younger than controls. Children from multilingual home environments were not evenly distributed: the SEMLA group consisted of 53% multilingual children, compared to 27% in DIL and 22% in the control group. For SES, there were significant differences between all groups and for preschool time, children in the control group spent significantly more time at preschool than the children in SEMLA.

One-way ANOVAs were conducted to compare SEMLA, DIL and the control group with regard to age, SES, and hours per week at preschool. Age differed significantly between groups, *F*(2) = 3.291, *p* = 0.039 (*n* = 417). A Tukey post hoc test revealed that children in DIL were significantly younger (*M* = 61, *SD* = 7 months, *p* = 0.034) than children in the control group (*M* = 63, *SD* = 7 months). There was no statistically significant age difference between DIL and SEMLA or between SEMLA and the control group. For SES, there was a significant difference between groups, *F*(2) = 13.45, *p* < 0.001. A Tukey post hoc test showed that SEMLA and DIL differed significantly with regard to SES at *p* = 0.043, SEMLA and control differed significantly at *p* < 0.001 and DIL and control differed significantly at *p* = 0.01. For current time at preschool, there was a significant difference between groups, *F*(2) = 3.379, *p* = 0.035. Children in the control group spent significantly more time at preschool (*M* = 38.71, *SD* = 5.52) than the children in SEMLA (*M* = 36.82, *SD* = 6.64, *p* = 0.039). For current time at preschool, there was a significant difference between groups, *F*(2) =3.379, *p* = 0.035. Children in the control group spent significantly more time at preschool (*M* = 38.71, *SD* = 5.52) than the children in SEMLA (= 36.82. *SD* = 6.64, *p* = 0.039).

### Preschool quality, ECERS-3

To estimate preschool quality, the Early Childhood Environmental Rating Scale (ECERS-3) [37] was used. ECERS is an internationally established tool for measuring preschool quality and has been more predictive of children’s learning than factors such as group size and staff-to-child ratio [87].^7^ ECERS third edition measures 35 items organized into six different subscales: Space and furnishing, Personal care routines, Language and literacy, Learning activities, Interaction, and Program structure. Although not adapted for the cultural context of Sweden, the rating-scale is considered to hold for international comparison [92]. The assessment was conducted by trained researchers, not involved in the project in any other sense and blind to the interventions and the aims of the study.

### Procedure

The preschools assigned to SEMLA (socioemotional and material learning) or DIL (digital individual learning for body and mind) had introduction courses prior to the pretesting. For SEMLA the introduction consisted of four 3 ½-hour evening sessions where the teachers were guided through the SEMLA intervention, their own part in the implementation and how to work with the children during the SEMLA sessions. SEMLA should be applied four days a week for approximately 1 ½ hours each day during the 6 weeks of intervention. For DIL the introduction consisted of four evening sessions of two hours where the educators were introduced to the Magical Garden digital game and learnt how to implement the game and support the children when needed. They were also taught the body-and-mind exercises and how these should be used. DIL was implemented one hour/day during the six-week intervention. The control preschools did not have specific training but met on one occasion for information about the self-evaluative toolkit, BRUK [68], administered by the Swedish National Agency for Education [69]. The control preschools agreed to work on the strand that concerned the learning environment and were then instructed to work with this instrument on their own and compare experiences afterwards, as a way to heighten their motivation during the intervention period (see [70]).

To support implementation, both SEMLA and DIL preschools had researchers or supervisors instructed to supervise the interventions. The teachers were also equipped with forms on which they were encouraged to follow children’s activities related to the intervention, and which further aided the staff in implementing the practices (see Additional files 1 and 2).

Following the evening instruction classes for the enrolled preschool staff, 2 weeks of pretesting of the children commenced at the preschools. The test situations were video recorded using Canon XA 10 video camera and for audio recording Sennheiser MKE 2 lapel microphones were used. All language and communication data from interaction and narrative come from these recordings. The videos were transcribed using the ELAN Video Annotation Software [93] by the first and third author and trained research assistants.

### Implementation fidelity

Fidelity of the implementation was tracked somewhat differently depending on the intervention. Preschool staff tracked how many days a child had been offered 1 ½ hours of SEMLA work. In the DIL implementation, each child’s frequency data and play time on the Magical Garden was registered in the device whereas the amount of body-and-mind exercises was registered in a log book describing which children participated, which activities had been undertaken and whether anything out of the ordinary had occurred. The mean number of sessions and standard deviation are reported in the results section. As described in Gerholm et al. [48], a standardized fidelity score was also calculated for both SEMLA and DIL. For SEMLA this score was based on the number of SEMLA sessions each child participated in. The calculation for the DIL intervention consisted of the standardized sum of the number of body-and-mind sessions and the number of Magical Garden sessions, weighted according to the mean play time for each child. For the children in the control group, zero was used as a fidelity score. This resulted in a standardized fidelity score with a mean of zero and a standard deviation of 1, where zero were treated as a baseline value.

For SEMLA, which did not depend on a strict script in the same manner as DIL’s game logs, a further fidelity measurement regarding the pedagogical quality was developed based on ratings using the extensive video data. All in all, 20 h of video recordings were retrieved from the SEMLA sessions, over the six-week intervention period at the nine units. The recordings were rated by one of the researchers using criteria based on the SEMLA documentation form describing and exemplifying how the seven components^8^ were to be implemented (see Additional file 1). Each of these components was operationalized to comprise four to eight different criteria, making an evaluation of 41 criteria per film. The conditions for reaching good/excellent fidelity can be summarized as the teacher’s ability to be responsive, not only to the learning group as a whole, but also to the individual children as a part of a collaborating team. To reach a good or excellent quality, the teacher was expected to often or routinely supply creative materials and to scaffold individual children with questions and comments, as well as with information and facts that enhance emotional desire, curiosity, reflection and learning, while exploring a problem as part of a learning group. The SEMLA ratings mirror the structure of the preschool quality environmental ECERS scale [37], where insufficient is rated from 1 to 2, minimal 2–4, good 4–6 and excellent 6–7.

In addition, all the project’s preschool units were visited at random intervals by three research assistants blind to the interventions, with instructions to video record five minutes of preschool activities (so-called “fidelity filming”). The purpose of the recordings was to give a glimpse of the daily practices at the different preschools and their potential tendency to practice a particular pedagogical agenda regardless of intervention or control assignment. This was conducted as a precaution in order to control for a SEMLA or control intervention preschool regularly using digital tablets training math or vice versa. These recordings were rated by a blind research assistant using a protocol developed for this purpose.

### Measures

The outcome measures included in the study were language, communication, math, executive functions, and socioemotional comprehension (see [48] for detailed descriptions). These were assessed in the following way: see (Table 2)

**Table 2:** Tests overview. All tests used pre- and post-intervention, and the targeted skills measures

Test	Skills measured
Language:	
The Peabody Picture Vocabulary Test [94]	receptive vocabulary
The Bus Story Test [95, 96] – used at pretesting	lexical diversity (number of word types used); information score (how many events a child included in the narratives), syntactic complexity (number of subordinate clauses), morphological complexity (amount of well-formed utterances), and text length (total number of clauses)
Frog, Where Are You? [97–99] – used at post-testing	lexical diversity (number of word types used); information score (how many events a child included in the narratives), syntactic complexity (number of subordinate clauses), morphological complexity (amount of well-formed utterances), and text length (total number of clauses)
What’s Wrong Cards [100]^a^	productive vocabulary, observation skills and created in order to develop emotional literacy
Communication^b^:	
An adapted version of ADOS [101]	meeting of gaze, adequate use of gestures, at ease body behavior, fluency/prosodic traits, following instructions, turn-taking behavior, and taking initiative/showing curiosity
Executive functions:	
The Dimensional Change Card Sort task (DCCS [59, 102])	cognitive flexibility/attention shifting (possibly working memory as well)
The Flanker Fish Task [103–105]	inhibition
The Head-Shoulder-Knees-Toes (HSKT, [106])	inhibition, focused attention, and working memory
Forward and Backward Digit Span [107]	short term memory, storage capacity, working memory
Auditory selective attention was measured using event related potentials (ERPs) to attended and unattended probe sounds embedded in stories, i.e. the Swedish AUDAT paradigm	ability to attend to one story while ignoring another simultaneously presented story
Emotional Comprehension:	
Test of Emotion Comprehension [108, 109]	socioemotional comprehension, ability to recognize facial expressions (drawn faces) of emotions related to different stories read to the child by the test leader
Math:	
An adapted version of the Number Sense Screener [110–112]	one-to-one correspondence, number sense cardinality, ordinality and subitizing

*a*. Note: What’s Wrong Cards were used as an additional method to assess verbal skills in the child. Each child watched three different cards depicting odd situations, such as someone trying to put a sweater on as trousers or ironing a hat, and were encouraged to describe the picture and elaborate on the peculiarities of the activities seen. However, as this did not yield enough data and we already had speech samples from the narrative task, we did not proceed to analyse the results

*b*. Note: In the planning of the study [48], communication was regarded as a composite measure including the novel communication-rating of video-filmed interactions and the emotional comprehension test, TEC. However, as we did not know what to expect from the novel measure used, in the analysis phase we decided to keep the two measure separate and abandon the composite

Most of the tests were behavioral standardized tests or adaptions based on standardized tests. For a subset of the children we also included Swedish AUDAT, an adaption of the experimental paradigm used by Neville et al. [4] to assess auditory selective attention with ERPs. The paradigm has proven sensitive to intervention effects in young children [4].

### Testing procedure

The pretesting of the children commenced two weeks prior to the intervention start and the post testing followed directly after the intervention. Trained research assistants (speech-language pathologists, psychologist, and social scientists hired for the project) came to the different preschools and conducted the testing in a secluded room, chosen by the preschool. The testing sessions were divided into two for both pretesting and post testing, each session being approximately 30 min. This was done to avoid fatigue and boredom on the part of the children. The order of the tests was: DCCS, TEC, Bus Story (pretest)/Frog Story (posttest), math, HSKT for the first sessions, and: Flanker, What’s Wrong Cards, PPVT, Digit span for the second session. The order was chosen based on a pilot study (Tonér & Gerholm, Language and executive function in Swedish preschoolers: a pilot study, under review, Applied Psycholinguistics). The sessions were video recorded in order to provide data on language and communicative behavior but also in order to check fidelity in test assessment.

Auditory selective attention was assessed through the Swedish AUDAT ERP-paradigm and could not be carried out on the complete sample. Thus, a subgroup of children was sampled to participate in the EEG-testing using a randomized priority list. Children and their guardians were previously informed about the general purpose and outline of the experiment and guardians had given informed consent about participation. Children were asked if they were ready and willing to record based on the order of the randomized priority list. If they declined, the next child on the list was asked. In the recording room they were seated on a small chair in front of a laptop (≈100 cm from the head) with speakers on each side (≈70 cm from the head). They were instructed on what participation would entail, and electrodes and a cap were applied. In Swedish AUDAT probe sounds are embedded in two simultaneously presented stories. The stories were differentiated by content, by gender of the voice of the reader, and by presentation to the left or right. The child was instructed to attend to one story while ignoring the other. Illustrations from the attended story were presented on the laptop. Probe sounds where either the syllable ‘Ba’ or a noise ‘Bzz’. The ‘Bzz’ was constructed by splicing 20 ms segments of the ‘Ba’ sound and scrambling all segments except the first and last. Both probes were 200 ms and presented randomly with respect to probe type, left or right presentation and inter stimulus intervals of 200 ms, 550 ms or 1000 ms. Each recording session involved two pairs of stories, one longer (7 min) story pair and one shorter (5 min) story, with comprehension questions after each story. A child participating in both pre and post session would hear 8 stories, and attend half of them, balanced over presentation to the left or right and with regard to female or male voice, and presentation order. EEG was recorded using a BioSemi (BioSemi, Inc.) activeTwo amplifier with 16 head channels and a CMS/DRL loop in a cap, two external mastoid channels and four external eye channels (for activeTwo and CMS/DRL details see http://www.biosemi.com/). All processing was done in EEGLAB [113]. Sampling rate during recording was 2 kHz, downsampled to 256 Hz offline, re-referenced to average mastoids and filtered using the “pop_eegfiltnew” function in EEGLAB with a pass band of 0.1 Hz and 40 Hz. Bad channels among the head electrodes were identified visually and interpolated (on average 0.06 electrodes in each pre or post recording). The data was epoched from a 100 ms pre-stimulus baseline before any probe sound to 500 ms post stimulus response. Artifacts, including ocular artifacts, were rejected automatically (epochs with head channel amplitudes larger than + 200/− 200 μV or eye channel amplitudes larger than + 100/− 100 μV in a moving time window of 200 ms were rejected) and based on visual inspection. An estimated 50% of the epochs were rejected, leaving on average 158 epochs per participant in each condition (attended/unattended) and session. This is 82% of the number of trials in Coch et al. [114] when testing older children (6–8 years), and 42% of the number of trials for 3–8 year olds in Stevens [115], both using the original AUDAT paradigm. The high rejection rate is unfortunate but in some respects compensated by our very high number of child participants, and two recording sessions. Thirty pre-intervention recordings and twelve post-intervention recordings were excluded due to noisy or flat average response or less than 100 epochs remaining for attended or unattended events after artifact rejection. Sixteen more pre-intervention sessions and four post-intervention sessions were excluded due to failed comprehension tests. For statistical analysis, 89 pre-intervention and 89 post-intervention participant sessions, were used, with 76 participants having both pre and post recordings.

### Reliability

With regard to the ratings of communication based on video recordings of the test session, a subset was scored for inter-rater agreement. Nonparametric tests were used and the overall correlation between raters was .82 (*p* < .001). With regard to inter-rater agreement for transcriptions, a subset of stories was transcribed by two annotators and the scoring based on the two versions was compared. For word types, syntactic complexity, number of clauses and well-formed utterances, scoring was identical for the transcriptions from different transcribers. For information score, the difference was at maximum two points.

### Background variables

The information gathered through questionnaires delivered to the parents consisted of the following information: socioeconomic status (SES), estimated (if possible) on the bases of both caretakers’ income and educational level^9^; the Swedish Communicative Development Inventory [72, 116]; age measured in months, as well as age at preschool start and number of hours per week spent at preschool at the time of the intervention; sex, which was included as a variable based on prior research in various areas [44, 76, 79, 117, 118]; second languages spoken and information on the child’s strongest language; information on developmental disorders and family history of language disorders; and the Strengths and Difficulties Questionnaire (SDQ), [119–121].

### Analytic strategy

The nested type of data in our study and the large number of measures, some continuous and some categorical, present challenges to statistical analysis. A type of analysis that is recommended for data with a nested structure and that can handle many variables of different types is mixed models [122]. Our planned analysis was a series of univariate mixed regression models described in [48], and below. The nested structure of individuals, preschool units and preschools was modeled using so-called random variables [85]. Because of an underestimated problem with collinearity, we also present an explorative analysis that combines the series of univariate models into one multivariate model. Aside from the planned univariate analyses and the exploratory multivariate analysis, we present correlations and group mean comparisons where some are planned, and some are exploratory, as stated in the text. The ERP measure *selective attention difference* was computed and analyzed as planned, except that only six frontal electrodes were used. We also added an ANOVA that was not described in Gerholm et al. [48] to test for differences between unattended and attended responses directly, and a similar ANOVA to test an unexpected late effect.

## Results

The main purpose of the current study was to investigate potential intervention effects of the interventions SEMLA and DIL compared to a business-as-usual control group. The results section starts with a planned univariate regression analysis [48] that did not indicate any such intervention effects. Then follows an analysis of collinearity and a multivariate analysis that is motivated by collinearity. After this, the selective attention results are presented, and then results regarding implementation fidelity and an explorative analysis of intervention group differences. Ending the results section is an overview which sums up the results thematically.

### Planned regression analysis

The planned regression models have been used to investigate the association (linear relationship) between one of the post-intervention outcome variables language post, communication post, EF post, TEC post or math post and a set of predictors comprising pre-intervention scores of the variables, intervention, individual background variables (sex, SES, SCDI, SDQ, age, preschool start time, L2, best language, and family language problems (FLP)), the control variables ECERS and fidelity, as well as interactions between pre score of the predicted variable and intervention, SES and intervention, and ECERS and intervention (PRE_SCORE×INTERVENTION, SES × INTERVENTION, ECERS×INTERVENTION). In the regression equation below the outcome variable (one of language, communication, EF, TEC, or, math) is denoted as POST_SCORE. The variable PRE_SCORE represents the same variable pre-intervention. X_g_, l = 9,…,17, represent background control variables (sex, SCDI, SDQ, age, preschool, start time, L2, best language and FLP). POST_SCORE_ijk_ refers to the response for the *i*th child, nested within *j*th preschool unit, in *k*th preschool.

POST_SCORE_ijk_ = α_jk_ + α_k_ + β_1_INTERVENTION_jk_ + β_2_SES_ijk_ + β_3_PRE_SCORE_ijk_ + β_4_FIDELITY_ijk_ + β_5_ECERS_jk_ + β_6_(PRE_SCORE_ijk_ × INTERVENTION_jk_) + β_7_(SES_ijk_ × INTERVENTION_jk_) + β_8_(ECERS_jk_ × INTERVENTION_jk_) + β_g_X_g_ + ε_ijk_, ε_ijk_ ~ N(0, ${\sigma}_{\varepsilon}^2$), α_j_ ~ N(0, ${\upsigma}_{\upalpha \mathrm{j}}^2$), α_k_ ~ N(0, ${\upsigma}_{\upalpha \mathrm{k}}^2$).

The equation above is a general model used for testing the hypotheses based on research question 1 and 4 (see also [48]). However, the intervention interactions in the model were non-significant in all planned regressions and were therefore omitted. This reduced the model’s degrees of freedom from 20 to 14. A minor correction of the Gerholm et al. [48] equations is that ECERS is modelled on the *j*th level instead of the *k*th level.

**Fig. 1:**
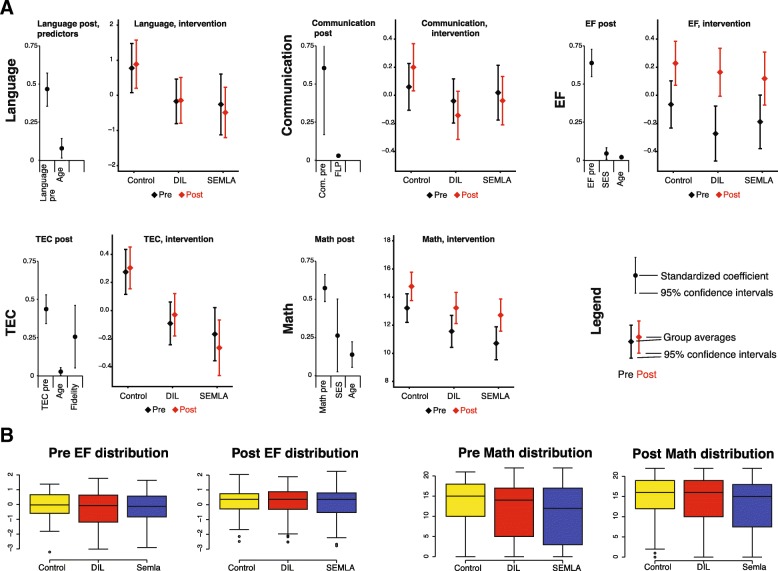
**a** Significant predictors of all outcome variables, with standardized coefficients and 95% confidence intervals. Also group averages pre and post for all outcome variables with 95% confidence intervals. **b** Distributions of EF and math, pre and post as quartiles

The models and their significant predictors are presented in Table 3 and in Fig. 1. The full models are presented in Additional file 3.

**Table 3:** Univariate regressions. Univariate regression results for each outcome variable. All significant effects are presented with regression estimates. Non-significant intervention effects are also presented. Auditory selective attention is presented separately (see Table 6: Selective attention regression). *P* values for estimates are omitted since they are exactly the same as for the main effects.

Selected results, main effects				Significant predictor estimates		
*Outcome variable*	*Predictor*	*DF*	*p*	*Estimate*	*SE*	*t*
Language post						
*Model DF = 14, error DF = 290,* *R*^*2*^ *= 0.319*	Language pre	1	<.0001	0.459	0.054	8.43
	Age	1	0.014	0.079	0.032	2.48
	Intervention	2	0.318			
Communication post						
*Model DF = 14, error DF = 302,* *R*^*2*^ *= 0.371*	Communication pre	1	<.0001	0.597	0.052	11.59
	FLP	1	0.020	0.030	0.013	− 2.34
				(FLP = 1, vs FLP = 0)		
	Intervention	2	0.131			
EF post						
*Model DF = 14, error DF = 259,* *R*^*2*^ *= 0.636*	EF pre	1	<.0001	0.629	0.045	13.95
	Age	1	0.001	0.020	0.006	3.32
	SES	1	0.024	0.044	0.020	2.27
	Intervention	2	0.179			
TEC post						
*Model DF = 14, error DF = 326,* *R*^*2*^ *= 0.368*	TEC pre	1	<.0001	0.431	0.047	9.16
	Age	1	0.034	0.027	0.013	2.13
	Fidelity	1	0.014	0.253	0.103	2.46
	Intervention	2	0.073			
Math post						
*Model DF = 14, error DF = 326,* *R*^*2*^ *= 0.565*	Math pre	1	<.0001	0.571	0.044	12.91
	Age	1	0.001	0.140	0.042	3.31
	SES	1	0.028	0.264	0.120	2.2
	Intervention	2	0.892

#### Multivariate regression model

Correlations among the post scores were investigated (see Table 4) and since there was a strong association between responses, we decided to conduct a multivariate analysis. In the multivariate analysis the effect of covariates is investigated on several response variables (language post, communication post, EF post, TEC post, math post) simultaneously and tested as a MANOVA.

**Table 4:** Pearson Correlation Coefficients, (Number of Observations). Correlations among outcome variables

	Language post	Communication post	EF post	TEC post	Math post
Language post	1	0.37***	0.40***	0.41***	0.36***
	(382)	(382)	(354)	(382)	(382)
Communication post	0.37***	1	0.06	0.20***	0.17**
	(382)	(396)	(357)	(394)	(394)
EF post	0.40***	0.06	1	0.38***	0.63***
	(354)	(357)	(365)	(365)	(365)
TEC post	0.41***	0.20***	0.38***	1	0.44***
	(382)	(394)	(365)	(404)	(404)
Math post	0.36***	0.17**	0.63***	0.44***	1
	(382)	(394)	(365)	(404)	(404)

Note: *p < 0.05 **p < 0.001 ***p < 0.0001

**Y**_ijk_ = α_jk_ + α_k_ + β_1_INTERVENTION_jk_ + β_2_SES_ijk_ + β_3_PRE_SCORE_ijk_ + β_4_FIDELITY_ijk_ + β_5_ECERS_jk_ + β_6_(PRE_SCORE_ijk_ × INTERVENTION_jk_) + β_7_(SES_ijk_ × INTERVENTION_jk_) + β_8_(ECERS_jk_ × INTERVENTION_jk_) + β_g_X_g_ + ε_ijk_, ε_ijk_ ~ N(0, Σ), α_j_ ~ N(0, ${\upsigma}_{\upalpha \mathrm{j}}^2\mathrm{I}$), α_k_ ~ N(0, ${\upsigma}_{\upalpha \mathrm{k}}^2\mathrm{I}$).

**Y**_ijk_ denotes the response vector with five components: language post and communication post, EF post, TEC post and math post. PRE_SCORE represent the same variables pre-intervention (language pre and communication pre, EF pre, TEC pre and math pre). X_g_, l = 9,…,17, represent background control variables (sex, SCDI, SDQ, age, preschool, start time, L2, best language and FLP). As in the univariate analysis, all interactions with intervention were non-significant and omitted from the model. Significant effects and non-significant intervention effects are tested using MANOVA, and significant predictors are presented in Table 5. All results are presented in Additional file 3.

**Table 5:** Multivariate Analysis of Variance, and estimates. MANOVA analysis of multivariate effects, and univariate regression estimates for significant predictors in the multivariate model. Significant MANOVA results and a non-significant effect of intervention are presented. Estimates are shown for all significant predictors for each outcome variable

Multivariate effects (selected results)					
*Predictor*	*Wilks’ Lambda*	*Num DF*	*Den DF*	*p*	
Language pre	0.853	5	229	<.0001	
Communication pre	0.689	5	229	<.0001	
EF pre	0.671	5	229	<.0001	
TEC pre	0.774	5	229	<.0001	
Math pre	0.787	5	229	<.0001	
Intervention	0.942	10	458	0.186	
Estimated effects for the multivariate model					
*Outcome variables*	*Predictor*	*Estimate*	*SE*	*t*	*p*
Language post					
	Language pre	0.397	0.066	6.050	<.0001
	EF pre	0.683	0.326	2.100	0.037
Communication post					
	Communication pre	0.592	0.059	10.040	<.0001
	Language pre	0.004	0.002	2.560	0.011
	TEC pre	0.009	0.003	2.540	0.012
EF post					
	EF pre	0.532	0.056	9.540	<.0001
	Communication pre	−0.877	0.409	−2.150	0.033
	Math pre	0.027	0.009	2.990	0.003
TEC post					
	TEC pre	0.440	0.056	7.890	<.0001
	EF pre	0.389	0.130	3.000	0.003
Math post					
	Math pre	0.464	0.061	7.540	<.0001
	EF pre	1.586	0.386	4.100	<.0001

### Auditory selective attention

**Fig. 2:**
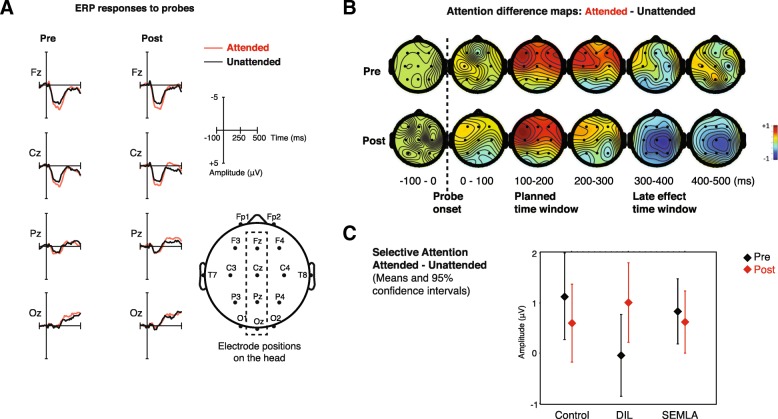
**a** ERP grand average responses on midline electrodes (Fz, Pz, Cz and Oz) for attended and unattended responses, pre and post intervention. **b** Topographic grand average plots of the difference between attended and unattended responses averaged over 100 ms intervals. **c** Mean difference attended - unattended, per intervention group, pre and post with 95% confidence intervals in the 100-200 ms time window

The auditory selective attention effect is a hypothesized difference between unattended and attended event-related responses in average amplitude 100–200 ms after probe onset. These latencies capture the broad positive peak that is typical in children’s responses to sounds, they are consistent with previous literature using AUDAT [4, 114, 115] and with our unpublished pilot data. The average amplitude for each participant was analyzed with an ANOVA with variables attention, electrode position, intervention and time (pre or post intervention). The results are presented in Table 6. There was a main effect of attention, and also an interaction between attention and electrode position, reflecting a stronger attention effect in fronto-central electrodes. There was no interaction between attention, time and treatment, and thus no intervention effects on selective attention. There were effects of electrode position, which is commonplace in ERPs but of little interest, and an interaction between electrode position and intervention that might have limited relevance as an indication of general group differences but is not analyzed further here. ERP responses are presented visually in Fig. 2a and b. Further ERP plots, grand averages of pre and post, for all participants, and all intervention groups can be found in Additional file 4.

**Table 6:** Auditory Selective attention results. A summary of ERP results regarding auditory selective attention. First, significant results from an ANOVA analyzing the attention effect at 100-200 ms is presented, and also the critical but non-significant Attention×Time×Intervention interaction. Second, two non-significant predictors of the selective attention difference are presented for comparison with similar regressions in Table 3. Third, selected exploratory correlations are presented. The last part presents exploratory ANOVA results for the late 300-400 ms attention effect, significant effects, and relevant non-significant effects

Attention effect ANOVA (selected results)			*Num* *DF*	*Den* *DF*	*F*	*p*
Attention			1	1606	6.3	0.0122
Attention×Time×Intervention			2	1606	1.33	0.2653
Electrode position			3	19000	1201.85	<.0001
Attention×Electrode position			3	19000	33.23	<.0001
Intervention×Electrode position			6	19000	16.62	<.0001
Selective attention regression (selected results, compare Table 3)						
*Outcome variable*		*Predictor*	*DF*	*p*		
ERP post		ERP pre	1	0.068		
*Model DF = 14*		Intervention	2	0.305		
*error DF = 66,*						
*R*^*2*^ *= 0.14*						
Selective attention, Pearson Correlation Coefficients (selected results)						
	Selective attention pre	*N*		Selective attention post		*N*
Language pre	0.23*	84		0.07		89
Language post	0.03	82		0.00		86
Late time window attention effect ANOVA (selected results)		*Num DF*	*Den DF*	*F*		*p*
Attention		1	1613	5.52		0.0189
Attention×Time×Intervention		2	1613	0.1		0.905
Electrode position		3	19000	321.42		<.0001
Attention×Electrode position		3	19000	0.75		0.523
Intervention×Electrode position		6	19000	16.62		<.0001

Note: *p < 0.05

A selective attention variable was then created using mean difference between attended and unattended responses over the six most frontal electrodes (where the effect was maximal in the ANOVA). This selective attention measure was created to fit regressions of the same form as for other outcome measures, and like them was analyzed in planned univariate regressions and in an exploratory multivariate regression, however with much lower number of participants (*N* = 81). These ERP-specific selective attention regressions did not reveal any significant effects of intervention, background variables or other variables, and the auditory selective attention difference was not a significant predictor of other outcomes. A few non-significant results are presented in Table 6 for comparison with other univariate regressions.

There were some unexpected ERP results: selective attention correlated with language in pre-sessions (see Table 6). In the group averages we also found a negative attention difference in a later time window (maximal at 300–400 ms) with a less frontal topography compared to the expected positive, early (100-200 ms) and frontal attention effect. This effect was potentially interesting since attention effects among older children and adults are often negative at longer latencies [123]). While the effect was nominally stronger in the post intervention recordings (see Fig. 2b) the analysis showed only a main effect of attention (see Table 6) with no interactions with time of test or electrode position. As in the ANOVA of the early attention effect there were also two less interesting effects, presented in Table 6: a main effect of electrode position and an interaction between electrode position and intervention. Since this late attention effect was unexpected and did not have any intervention effects (see Table 6) it is not explored further here.

### Implementation fidelity

In the regressions, fidelity was a normalized value based on number of sessions each child attended and also, in DIL, time spent with the game Magical Garden. While thought of as a control variable, fidelity predicted TEC (see Table 3). To make further results more accessible we will discuss implementation fidelity in terms of number of sessions.

In SEMLA, children attended on average 13 sessions (*SD* = 4.6), while instructions prescribed 24 sessions in total. The range of sessions per child was 10–25, indicating that the low average was not a result of a few outliers. Each session was about 1.5 h. In the DIL intervention average number of sessions was 20.4 (*SD* = 4.6, range 10–28) for Magical Garden and 19.7 for body-and-mind (*SD* = 4.5, range 9–28). DIL sessions included both types of sessions, but participation could vary as seen in the slightly different averages. The instructions prescribed 20–30 sessions. Body-and-mind sessions were about 15–20 min, and average Magical Garden sessions were 27 min.

Implementation fidelity of SEMLA was also assessed by structured quality ratings of video material. The quality ratings of SEMLA show that only one unit reached the level of excellent with a score of 6.7. Three units varied from 4.1 to 5.1 and reached “good”, two varied between 2.6 and 3.9 were rated as “minimal”, and one unit was rated to reach an “insufficient” quality at 1.2. Similar video ratings of DIL implementation fidelity was not considered relevant since this intervention was more scripted.

### Intervention group differences

In order to find any nuances or trends of interest that could help us understand the general results, we explored intervention group differences with a series of one-way ANOVAs and Tukey post hoc tests. The control group scored better on several measures compared to the intervention groups. In math, control scored better than SEMLA both pre and post intervention (See Fig. 1): Pre intervention differences were significant (*F*(2) = 4.853, *p* = 0.008), as were post intervention differences (*F*(2) = 3.499, *p* = 0.03). Post intervention scores for language were lower in SEMLA than in the control group (ANOVA: *F*(2) = 4.114, *p* = 0.02; Tukey post hoc test: *p* = 0.014), and post scores for communication were lower in DIL compared to controls (*F*(2) = 4.114, *p* = 0.02). Post intervention scores for language were lower in SEMLA than in the control group (ANOVA: *F*(2) = 4.114, *p* = 0.02; Tukey post hoc test: *p* = 0.014), and post scores for communication were lower in DIL (*F*(2) = 4.114, *p* = 0.02).

Ratings of preschool quality using ECERS-3 also differed significantly between groups (*F*(2) = 68.36, *p* < 0.001). A Tukey post hoc test revealed that preschool quality was higher in control than in SEMLA (*p* < 0.001) and higher in the control group than in DIL (*p* < 0.001). There was no significant difference between the two intervention groups (*p* = 0. 997). Units within the same preschool differed substantially in their ratings.

### Results overview

#### Regression results overview

In both univariate and multivariate regressions, all post-intervention measures were significantly predicted by pre-intervention measures of the same variable. Age predicts post intervention performance in language, EF, TEC, and math in the univariate analysis. SES predicts post EF and post math in the univariate analysis, likewise fidelity is a significant predictor of post TEC. Presence of family language problems (FLP) negatively predicts post communication.

In the multivariate regression there were no significant effects of background variables such as age, SES or FLP; however, pre-intervention scores for language, communication, EF, TEC, and math all have significant effects on post intervention scores: EF is predicted by pre-scores for math and communication, the latter negatively related. Math, language and TEC are all predicted by EF. Communication is predicted by language, and TEC; (see Table 5). We take the differences between univariate and multivariate analysis to reflect the relatively strong collinearity between many outcome variables (see Table 4, and Table 5), compared to the significant but weaker effects of the background variables age and SES (see Table 3).

#### Intervention effects

In both planned univariate regressions and the follow up multivariate regression, there were no effects of interventions, neither as direct predictors nor as interactions. In the univariate regression model for communication, the interaction ECERS×Intervention was significant when other non-significant interaction factors were present in the model. However, when non-significant interaction predictors were removed, ECERS×Intervention was no longer significant and was removed as well. See Additional file 3 for details of non-significant results. The raw differences between intervention groups were small. The largest positive difference compared to controls was EF in the DIL group. EF difference pre – post in DIL was 0.15 standard deviations larger than the same difference for controls. The present study is not designed for such small effects: the sample size needed to detect such small effects is > 350. In Fig. 2c, mean post selective attention for DIL, is outside the 95% confidence interval for selective attention post. This effect is 0.24 standard deviations in the frontal electrodes, a small effect according to Cohen’s rule of a thumb [126]. A sample size of 151 would be needed to detect such small effects. Our sample size was designed to handle medium to large effects, such as Neville et al. [4], were the effect size for one group, using the same paradigm, is 0.83 standard deviations among the best channels. Sample sizes in this section were calculated using G*Power [124]. The trend for an effect in ERP selective attention in DIL is discussed below but is not considered a genuine intervention effect.

The lack of intervention effects implies that there are no differences between effects, no mediating effects explaining the intervention effects, no moderating effects, and no differences in the distributions of intervention effects. The hypothesis about intervention effects (RQ1) found no support, rendering the hypotheses based on such an effect (RQ2, RQ3, RQ4 and RQ7) irrelevant.

#### EF

Only one hypothesized predictor of outcome variables was significant in the regression analyses. SES predicted EF in the planned univariate analysis. EF was also hypothesized to mediate intervention effects on language, communication, TEC and math. Math and language differences pre and post were also hypothesized as mediators of intervention effects in EF. While none of these mediating effects were present our results show that these variables are related both as correlations and as predictors in the multivariate regression (with the exception of language as a predictor of EF). Thus, EF pre-intervention predicted post-intervention language, TEC, math and (in a negative direction) communication. Math pre-intervention also predicted EF post intervention. EF is thus a predictor for most of the variables where it was hypothesized as a mediator for change. EF post also correlates with language post, TEC post and math post (see Table 4).

#### Age, SES, sex differences, multilingualism and time at preschools

Age predicts post-intervention performance in language, EF, TEC, and math in the univariate analysis. Age also correlated significantly with SCDI-words (Spearman’s *ρ* = 0.29, *n* = 383, *p* = < 0.001) and to SCDI-morphology (*ρ* = 0.23, *n* = 378, *p* < .001), showing that older children had higher language skills, as reported by parents. There were no effects of age in the multivariate analysis.

SES predicted EF in the univariate analysis. While average SES of the multilingual group was lower than monolinguals, the hypothesized relation between SES and language was not significant in the regressions. Hypothesized positive effects on EF due to multilingual background, negative effects on math from having another L1 than Swedish, and positive relationship TEC and language were all non-significant.

The hypothesized sex differences in communication, EF or TEC were not significant (see Additional file 3).

A Kruskal-Wallis test was used to examine potential differences between monolingual Swedish-speaking children and multilingual children with regard to age at preschool enrollment and SES. Mean age at preschool start was slightly higher (*M* = 19 months) in the multilingual group than in the monolingual group (*M* = 17 months), but the difference was not significant. There was a significant SES difference between groups (χ^2^ = 27.81, *p* < .001, df = 1) with higher SES for the monolingual group (median = 8, *n*_1_ = 264) than the multilingual group (median = 6, *n*_2_ = 129).

Based on results from our pilot study, it was hypothesized that age at preschool start would have a negative relationship to current time spent in preschool (measured in hours per week). Spearman rank-order correlation coefficients were computed and there was a significant negative correlation between age at preschool start and weekly amount of time at preschool (*ρ* = − 0.16, *n* = 390, *p* = 0.0015), thus indicating that children who were younger at preschool enrollment currently spend more time per week in preschool.

Higher SES was expected to correlate with children spending more time at preschool. There was a significant but small positive correlation between SES and weekly time in preschool (*ρ* = 0.1, *n* = 391, *p* = 0.046), thus indicating that children from relatively higher-SES backgrounds spend more time per week at preschool than children from lower-SES backgrounds. However, there was no significant correlation between SES and age at preschool start. There was no significant correlation between SES and SCDI-words (*ρ* = 0.05, *n* = 378, *p* = 0.32). There was however a significant correlation between SES and SCDI-morphology (*ρ* = 0.24, *n* = 378, *p* < 0.001).

#### Study limitations

There are some limitations to this study to be discussed. To begin with the available resources meant that the study was set to 6 weeks based on Neville et al.’s study [4], which showed results from a short-term intervention. However, Neville et al.’s study was two-generational and as such more comprehensive, involving both preschool and home. This suggests that future studies should be more comprehensive and implemented for a longer period of time in order to enhance the likelihood for significant effects. The 29 units were divided into three time-spans, which effected the randomization, as has been discussed already above. A limitation is also that this municipality is inhabited by a more than average amount of higher SES-families, and RCT:s are known to show effects mostly on lower-SES children, as explained by Wilson & Farran [32] among others. We therefore suggest that future studies in the Swedish context be situated in low-SES areas where learning potentials are expected to be greater. Another limitation in the context of intervention RCT studies, is that the involved preschools’ pedagogical quality was shown to be higher than average, something that the ECERS-3 evaluations confirmed. A limitation, also lifted by [125] can be that the interventions were “*simply not ready for trial*” (p. 258). Both interventions might be limited according to how well they were designed and performed as well as according to their strength and intensity. We suggest that future studies make use of more pilot testing and quasi-experimental designs, before undertaking a more largescale RCT in the search for generalizable evidence. Such preparatory studies should include investigations to make sure that the intervention components are functional in the particular context in which the intervention is implemented, that intervention preparations in terms of training of teachers are efficient and that the tests used to evaluate the study are valid and reliable in relation to the specific learning goals targeted in the interventions.

## Discussion

No statistically significant results were found in relation to effects of the two interventions on children’s language and communication, EF, socioemotional comprehension and early math (RQ 1–4, 7). The sizes of the behavioral intervention group differences are very small, below what is referred to as ‘small effects’ in Cohen’s rule of thumb [126] and below the effect sizes the study is designed to detect [48]. The discussion will first turn to possible explanations for this null result, followed by a closer discussion of the results and tendencies found in sub-parts of the data, e.g., the relation between background variables and outcomes on the one hand, and between different outcome measures on the other (RQ5 and RQ6).

### Interventions

The SEMLA intervention is based on principles which to some extent are part and parcel of the general approach in Swedish preschools, such as group-based collaboration with playful exploration of a common overarching problem or theme. The rationale behind SEMLA is that it was expected to impact children’s outcomes indirectly, for instance in that EF is enhanced by processes of verbal reflection and focused attention or that math is improved by children spending time with activities involving measuring, engineering and construction. DIL on the other hand, consists of individual, specific training of attention and early math skills and can thus be regarded as a contrasting working method compared to SEMLA. However, neither SEMLA nor DIL showed any effects on outcome measures compared to the control group, in which teachers and children carried on with business as usual in accordance with the preschool curriculum.

### Intervention implementation

Both interventions were implemented by the regular preschool staff, with support from researchers and assistants. In the present study, the learning objectives were made clear during the instruction classes prior to interventions for both DIL and SEMLA staff. However, due to the contrasting nature of the interventions, there were differences with regard to intervention complexity and the specificity of intervention guidelines/manuals. For DIL, there were detailed instructions for how to teach the body-and-mind exercises (Additional file 2), and for the digital tablet game Magical Garden, the instructions to the child were delivered consistently through the tablet. SEMLA, on the other hand, did not have to be identically implemented across preschool units, since teachers were free to implement the particular means of helping children progress towards the learning goals, guided by examples from the SEMLA documentation form (Additional file 1). With regard to level of teacher instruction, Bleses et al. [21] recently conducted a large-scale Danish preschool intervention study, targeting language and pre-literacy skills and comparing the effect of script-based versus open intervention strategies. When teachers were provided with clear goals to strive towards but were left to their own devices to reach these objectives, the success of the intervention was far greater than among the teachers who had to follow strict scripts for teaching. In light of the study by Bleses et al. [21], it could thus be noted that the success of an intervention may depend on the level of action space given to the teachers, but that it may also rely on the specificity of the goals to strive for. Whereas the current study investigated potential intervention effects on a vast array of skills, it may be advisable to have a narrower scope in future preschool intervention studies. Future studies are needed to clarify the role of script-faithfulness of the SEMLA and DIL methods, and more research is needed with regard to implementation fidelity and effectiveness of pedagogical methods that are open-ended and/or highly complex.

Previous studies have indicated that in order to achieve effects of interventions, the level of difficulty needs be continuously adjusted to each child. For Magical Garden in DIL, this was the case, since the game is adaptive and provides tasks according to the child’s ability and progression through the game. The body-and-mind exercises are harder to adapt individually, and it is unclear how this could have affected the intervention outcomes. The SEMLA intervention is individually adjusted in the sense that the teachers are expected to adjust to and to scaffold each child on his/her level. SEMLA was supervised and checked for implementation quality, but it is difficult to control for individual teachers fulfilling their part of the implementation. However, we have no reason to believe that the level of SEMLA was too high for the involved children.

### Intervention duration and fidelity

The duration of the intervention was set to 6 weeks. Initially, a longer intervention program was planned. However, previous research with a similar focus of interest and similar target groups has led to intervention effects after intervention periods of a similar duration as in the current study (e.g. [4, 104, 19]). It was furthermore deemed too intrusive to keep the preschools committed to the project for a full semester, with consequences such as not being able to follow other interests, go on excursions etc. Additional factors for the decision to have a six-week intervention period were time and funding available. It is possible that the kind of pedagogical methods included in the current project could have been more successful if the staff had had more time at their disposal. In particular, SEMLA could have benefitted from this, since some of the teachers expressed difficulties with getting into the prescribed activities (see Lenz Taguchi et al., forthcoming). SEMLA was more time-consuming and more demanding to implement than DIL, and the results regarding intervention fidelity reveal that SEMLA units did not fulfill the requirements regarding number of sessions. The mean exposure to SEMLA was 13 out of the prescribed 25 sessions, compared to the mean exposure in DIL, which was 20. Fidelity is crucial in intervention studies, but has been found to be rather low, even in studies with a high level of support and coaching from researchers e.g. [127–129]. However, DIL did not have an effect on the targeted skills although intervention fidelity was in line with recommendations. The body-and-mind exercises were based on a successful intervention program in Head Start classrooms [4]. It should, however, be noted that the efficacy of Magical Garden as a way of improving early math skills has not previously been evaluated beyond measuring children’s progress within the game itself.

### How do we measure progress?

The choice of test battery is crucial when it comes to intervention studies. The tests must target and assess the same skills that the interventions target, but at the same time, the test should not be too close to the intervention targets, as this would constitute training for the test. In this study, the results from pre- and post-testing in the total group of children show that the test results improve slightly with time and that the different measures correlate significantly at pre- and post-testing, indicating that the measures used were reliable. However, as the intervention groups did not improve more than controls, we must also conclude that the interventions were not better than business as usual. The connections between tests, what they measure, and the skills actually trained within a particular intervention or pedagogical practice are not always clear-cut. This was the case for socioemotional comprehension and communication, which were both hypothesized to improve more in SEMLA, which was thought to focus children’s abilities to be empathetic, listen to one another and pay attention to each other’s utterances and thoughts to a higher degree than DIL. However, this was not the case. There are several test tasks and measures that need further investigation with regard to validity and reliability, not least since they have not previously been used in the Swedish context. There is one result that stands out as particularly unexpected: DIL had improvement of early math skills as its primary target, through the application Magical Garden, and yet there was no improvement in early math skills in the DIL group. The math test was not based on this game, but the same type of mathematical calculations appeared in both the game and the math test. Why did the DIL intervention not succeed in improving these children’s math abilities above the level of the groups who did not train math in this specific and targeted way? Previous research has revealed a lack of far transfer with regard to computerized working memory training [29], but less is known with regard to math training. In a study by Goldin et al. [130], children showed transfer of EF skills after an intervention consisting of computerized games, but only when the assessment was also computerized, suggesting that changes in the contextual setting can hamper transfer of specifically trained skills. Another tentative explanation comes from a recent qualitative study in Swedish preschool by Nilsen [131], who suggested that children may not learn the intended content in a pedagogical application, but rather progress through a game by means of trial-and-error.

With regard to the measure of auditory selective attention, the ERP selective attention effect did not show any intervention effect in the regression analysis or ANOVA (see Table 6), but there was a small change in the DIL group (see Fig. 2c). Pre-intervention amplitudes were lower in the DIL group compared to both SEMLA and control but after intervention, amplitudes were similar. There is thus a problem with group differences before intervention, weakening any conclusions about an intervention effect. The effect size is also small, see discussion in the results section. Considering that DIL is in part based on training that has previously been shown to have effects on the same selective attention ERP measure [4], our results are not in sharp contrast to that research, but rather a weak tendency in the same direction. This trend is also in line with the notion that ERP effects are often sensitive to group level experimental manipulations but less stable over repeated tests of the same person, while many stable psychological tests are not very sensitive to experimental manipulations cf. [132].

### Future direction

Some additional questions arise in the context of the current study. What did children learn in the control group where business as usual was implemented? This is of particular interest since the control units had significantly higher preschool quality, as rated with ECERS-3, than the intervention groups. To what extent are preschool teachers effective in employing pedagogical strategies, whether these are advocated by their education, part of a research project or stem from ideological beliefs of child rearing and teaching? Given the rather ambitious goals of the Swedish preschool curriculum [69], it would be expected that preschool teachers have a high level of control of pedagogical means and how these means support individual development and learning. However, in light of a recent preschool audit by The Swedish Schools Inspectorate [133], revealing uneven preschool quality, this is something that needs further exploration. The present study is but a first step in building a scientific base from which to provide this knowledge for the Swedish context.

Apart from evaluating which of two pedagogical methodologies that were best suited to enhance different abilities in children, the study aimed to add to prior research by investigating and hopefully disentangling the relation between background factors like SES, age, sex, languages spoken and outcome variables. In addition, the study aimed to clarify the potential relations between the different outcome variables language and communication, EF, socioemotional comprehension, and early math. Below, we discuss the results and tendencies found in the data in relation to first, background factors, and then the relation between tested skills.

### Background factors

While prior studies have found a clear relation between intervention and enhanced executive functions in preschoolers from low-income backgrounds [12, 31], these results have been hard to replicate in more diverse SES samples [32]. The present study had a mainly higher-SES population, when SES is measured as a combination of parental education level (4 grades: elementary school, upper secondary school, vocational education, and college/university) and family income (3 grades: 0–200,000 SEK, 200,001–500,000 SEK, and 500,001 > based on both parents scores divided by two). However, going into details of the data, there was a bias as to the spread of SES between the groups, yielding a control group which had children with significantly higher SES than both the SEMLA and DIL groups. The DIL group, in turn, had a higher SES than the SEMLA group. Based on earlier findings, children with lower SES (in this case both intervention groups as compared to the control group) would be expected to improve more than the children with higher SES (e.g., the control group), at least in EF and auditory selective attention (e.g. [4]). As this was not the case, either our sample did not comprise enough low-SES^10^ children, or the interventions simply were not better than business as usual in enhancing the targeted skills. SES was also correlated to both EF and math, which was in line with previous research. Yet another complicating factor regarding SES in the present sample is that the children with lowest SES (most of whom were assigned to the SEMLA intervention) also formed the group with the highest proportion of multilingual children. As SEMLA is an intervention which in many respects relies on language use and interaction, this could have put this group at a disadvantage. Also, the testing procedure and, obviously, the results thereof, are challenging when the child is not fluent in the language of testing. A study with children from more diverse SES backgrounds, and from various parts of the country, would have given a better foundation for a study of this kind. Time and funding limits did affect the ambition, as did the preschools themselves: preschools with many lower-SES families, which in this setting also meant that they were less familiar with Swedish, would not have had the time needed to enroll in research of this kind, which demands quite a bit of devotion and time. So, biases are likely even in larger-scale studies, unless we find ways to make interventions less straining for the staff. A suggestion made by other research [135] is to try effects in small-scale, well-controlled, and highly supervised studies and only proceed to larger-scale contexts once teachers have proven that they fully understand the implementation part and the effect of the intervention is documented. This is worth pursuing but does not do away with the problem of potentially more complex pedagogical methodologies like SEMLA.

Lastly, in relation to SES, even high-SES children should benefit and enhance their abilities while in preschool, so the general finding that this group of children rarely shows effect in intervention studies is problematic (see however [33, 34] who found effects in high-SES children for pre-literacy intervention). Our understanding of why this group of children is difficult to further improve in regard to the targeted skills is low. Therefore, in order to fulfill the curriculum goal of offering a preschool for all children, this need to be addressed in future studies. Likewise, the findings of this study which also replicate earlier studies, is that SES is correlated to all outcome measures (language composite, communication, EF composite, TEC, math), again indicating the need for preschools to improve their pedagogical techniques in order to give all children an equal start in preparing for the school years to come.

Among the hypotheses was also one pertaining to bi- or multilingual children. While bilingual children have long been reported as having an advantage in terms of EF skills (in particular inhibition and flexibility), this belief was recently challenged. Duñabeitia et al. [136] conducted a large-scale study with school-aged children and adolescents and found no support for a bilingual advantage for inhibition. A recent meta-analysis did not reveal enhanced EF in bilingual adults [137]. In the current sample, there was a significantly higher proportion of bilingual children in the SEMLA intervention group compared to both the DIL and control groups. This is unfortunate but explicable, since children typically attend preschool in the area where they live, and low SES tends to come together with a multilingual background, leaving a particular preschool with a homogenous population [138]. This is also seen in that monolingual children in the sample had a significantly higher SES than the bi- or multilingual children. Thus, the low-SES and multilingual situation of at least one of the SEMLA intervention groups could have affected the outcome.

Time at preschool has been shown to influence children’s life outcomes, at least when the quality at the preschool is high (e.g. [36]). This led us to expect that children who started early and/or stayed longer each day could potentially benefit more from good pedagogical input than children who entered preschool at an older age and/or spent only a limited amount of time at the preschool. We did not find any such indications in the present data. What we could see was that if a child starts preschool early (e.g. around 1 year old), s/he will also spend longer days at preschool when s/he is between four and six-years old. In order to address the question of whether and how preschool attendance relates to life prospects, we would have to return to the sample in years to come. There was no correlation between SES and preschool start, but there was a tendency for higher-SES children to also spend more hours/week at the preschool.

However, one complicating factor in terms of similarity between groups (in line with earlier complications such as SES and multilingualism) is that the children at control preschools had a significantly greater presence (hours/week) at the preschool than the SEMLA groups. The difference between the control and DIL groups was not significant. The children in the DIL group were also significantly younger than the children in the control group, but not the children in the SEMLA group (in the SEMLA group, the age range was 49–74 months, in the DIL group 46–74 months and in the control group the age range was 44–74 months at pretesting) making the skewness of groups go through almost all background variables (the exception is sex where there was an even distribution between groups).

As for age, we expected that a higher age would correspond to higher scores in all areas tested. This is trivial in the sense that children develop, regardless of interventions, and can be expected to improve with age. This was also found to be the case, as age was correlated to all measures (language, EF, socioemotional comprehension and math skills) except communication. The measure of communicative ability was a novel invention of this project (Tonér & Gerholm, Language and executive function in Swedish preschoolers: a pilot study, under review, Applied Psycholinguistics). It was based on the screening tool ADOS [101], and targeted behaviors connected to interaction quality such as meeting of gaze, gestural behavior, adequate response to questions, etc. The many nonverbal aspects of the measure can explain why it did not follow language generally in terms of predictive value. Social and pragmatic ability is a skill that is unevenly spread in populations and even if it is highly malleable and might change with age, a very young child can easily outperform a much older child given that their interest in interaction and other people, their self-esteem, and general outgoingness differ. At the same time, mood and other more fluctuating aspects of behavior can influence how a particular child is rated, making the scores potentially unstable if used only twice as in the present data.

The Strengths and Difficulties Questionnaire (SDQ), a questionnaire that both preschool staff and the children’s parents filled in, was used to see whether specific aspects of personality traits would matter for the study outcomes. We found no such correlation, neither in regard to other background variables nor to the skills tested in the pre- and post-testing. There was further no difference between the groups as to SDQ.

As for EF, there were no differences between the groups at either pre- or post-testing.

Between the intervention groups, there were furthermore no differences in communication score at pretesting but at post-testing, the control group scored significantly higher than the DIL group. As there is no reason to assume that DIL would have had a negative influence on children’s pragmatic skills, this is not easily explained. Children were tested by the same test leader in the clear majority of cases (some exceptions can have occurred due to illness among testing staff) both pre and post, and a similar test-retest difference could be expected.

Yet another result that needs some footwork to account for is that the control group at pre-testing had better math scores than the SEMLA group. However, at posttest the difference was non-significant. It is unclear how this came about, in particular as our expectation of the SEMLA intervention was not particularly high in regard to math, which was elaborated on and practiced in a more holistic manner in comparison to DIL’s firmer math training. As SEMLA did not show intervention effects we cannot interpret this posttest finding as if SEMLA had effects on math. We furthermore have no reason to assume that children in the control group deteriorated in regard to math between pre- and post-testing. As already mentioned, the surprising finding in regard to math was that the DIL group did not enhance their skills.

Earlier research made us expect to see a language advantage in girls [39, 41, 139]. No such findings were evident from the data, nor did a pilot study on a similar group of children reveal any differences in language between girls and boys (Tonér & Gerholm, Language and executive function in Swedish preschoolers: a pilot study, under review, Applied Psycholinguistics). As recent evaluations of school performance and results in older children and adolescents [140, 141] show a clear advantage for girls, a comment from our study would be that either times are about to change and the generation of boys studied here will catch up with girls even later on; or, the gender-related difference seen in older children and adolescents does not appear until after the children have left preschool.

Preschool quality was a measure evaluated by ECERS-3 in the present study. Results from prior studies on preschool quality [e.g. 36, 38, 39] indicate that attending a high quality (as measured by ECERS mostly) preschool has long lasting effects in areas such as cognition, literacy and general school readiness. These studies were not short-term intervention projects, making comparisons flawed, yet the results of the present study show that preschool quality was significantly higher in the control preschools compared to both SEMLA and DIL preschools. Moreover, all but three preschool units (which were rated “minimal”) within the present study were rated from “good” up to “excellent”, making a distinction based on qualitative aspects less usable as a sorting variable. A curious finding is that the ECERS-3 team in some cases rated different preschool units within the same preschool very differently. In these cases, the units share the same physical space but occupy different rooms. In many cases the teachers also go between and cover for each other in the event of absences, etc. The quality would be expected to be the same. If the difference relates to specific teachers being in one unit rather than the other at specific times, the need to understand teacher impact on pedagogical practices in more detail is urgent. Another possibility is that different members of the ECERS team visited the different units and interpreted the findings differently. Future studies would have to proceed with a closer scrutiny of the relation between the ECERS-3 ratings scales and the pedagogical skills and working conditions of the teachers and rating teams.

Summarizing background factors, we can see that the skewness of the randomization led to the control group starting out with higher SES and longer days than the SEMLA group, which in turn had a large group of multilingual and lower-SES children. It cannot be ruled out that this influenced the study outcome and future studies will have to find ways to balance groups more evenly. Adding preschool quality to the mix, we see that the control group appears to have also been favored by the highest quality marks of the assessed preschools. As has already been mentioned, the current study was performed in three waves where each wave had to be randomized without information on how the following groups/preschools would be composed. This is a drawback that should be avoided in the future.

Fidelity of intervention was measured as the amount of time a child was involved in the intervention, the control group having the value 0. Our measure of socioemotional comprehension, TEC, was predicted by the fidelity of the intervention in the univariate analysis. Perhaps children with high socioemotional comprehension (as measured by TEC) are more in tune with teachers and other children and this resulted in higher participation? This remains highly speculative, and we have not found any further evidence in this direction. Most likely, it is a spurious effect, and we present it without further attempts at interpretation.

Although research supports the possibility of obtaining effects from interventions as short as five to eight weeks e.g. [104, 4] there is reason to discuss how realistic rapid change might be in the selected outcome measures. Complex skills like language, EF and socioemotional understanding share the problem of also being difficult to evaluate and assess, as these skills tend to blend and depend on one another and, potentially, on other skills that were not tested [142]. Adding to this, the standardized tests available for clinical use are often too time consuming and focused on children at risk to suit the research intervention context. In the present study, we further needed to test an array of complex skills within a limited time frame, which made the assessment even more delicate (Tonér & Gerholm, Language and executive function in Swedish preschoolers: a pilot study, under review, Applied Psycholinguistics). This stated the present study found pre-intervention measures to predict post-intervention measures in both the univariate and the multivariate regressions analyses, indicating that the measures per se were up to the task.

### Relation between outcome variables

As skills come together in complex ways, the results in some domains are expected to correlate more than results in other domains. This is also why a composite measure was used, e.g., for language on the one hand and EF on the other. The results showed a correlation between measures as expected. Furthermore, EF was predicted by pre-intervention scores for math, i.e. having a high/low score on the math tasks was related to the child’s scores on EF. EF was in general indicative of other measures; apart from math, it predicted language and TEC. This is likely a result of abilities being related to one another and to a background general cognitive ability measure (such as IQ, which was not tested in the present study). SCDI-III, our parental questionnaire measuring the child’s productive vocabulary and morphology, would similarly be expected to correlate with the language measures actually tested on the child him/herself, (such as PPVT and the morphosyntactic and semantic measures extracted from the narratives). Results from the post-testing show that both SCDI-words and SCDI-morphology correlated significantly with each other, PPVT, number of subordinate clauses, and the information score. However, less expectedly, neither SCDI-words nor SCDI-morphology correlated with the following measures, all extracted from the narrative data: number of unified predicates, the number of morphosyntactically well-formed utterances, and the communication score. SCDI-words and SCDI-morphology further differed in their relation to SES, as SES did correlate with SCDI-morphology but not with SCDI-words from the same questionnaire. Age and SCDI were, more expectedly, correlated for both words and morphology. One thing to keep in mind while investigating SCDI and other parental questionnaires is that parents tend to interpret questions differently. As for the morphology measure of SCDI-III, it can be difficult for parents to understand what is being asked when they are instructed to check the kinds of sentences their child uses most, guided by examples of utterances with or without, for example, subordinate clauses. However, as the word count part of the SCDI-III is fairly straightforward, one would expect a correlation with the word measure rather than with the morphology one.

Language is a complex skill composed of a number of different abilities, apart from also having both a productive and a perceptive side and being part of tests which also target EF, socioemotional comprehension, math, etc. As many intervention studies use either a single measure, such as vocabulary size, or a composite measure for language, the results from the present study will have to be used as a starting point for more detailed examinations and analyses of the different parts of language use and understanding and, in particular, the reliability and validity of the tests used to assess these different parts where cultural adaptation is a much needed aspect (Tonér & Gerholm, Language and executive function in Swedish preschoolers: a pilot study, under review, Applied Psycholinguistics).

The ERP attention difference, measuring auditory selective attention, had a positive correlation with language (pre-intervention) see Table 6. This possibly reflects general task demands such as listening to the story and communicating with testers, i.e. language skills might help children understand and execute the attention task, perhaps more so the first session, but this is a highly speculative explanation.

Another unexpected ERP effect was a late (300–400 ms) negative attention effect (see Table 6 and Fig. 2) with central topography. The effect is similar to attention effects in adults and was unexpected for the present age group [114, 143]. This effect seems stronger in post testing but the analysis shows an attention as a main effect that does not interact with time (pre or post session). The effect might be of interest when comparing our population with populations in previous research, but this is beyond the scope of the present paper.

### Novel rating system for communication

As stated above, the communication rating measure was novel and only tested in a pilot to the present study. In the present study it was not correlated to the other language measures, which was expected, as a child can be perfectly in tune interactionally despite not having a large vocabulary or complex syntactic abilities and vice versa. An indication that the measure is worth pursuing in further studies is that it was predicted by the background factor Family Language Problems. These problems could, of course, be of a strictly verbal nature (such as dyslexia) but they could also relate to more interaction-related difficulties such as autism spectrum disorders etc. Future studies will have to look into these relations more closely. Also, communication and EF were negatively related at pretesting. This could be explained by the fact that children who have difficulties with attention and with focusing on the testing tasks might also find it difficult to interact with the test leader. At post-testing there was no significant relation between the two scores, potentially due to children being more at ease with the test situation and/or test leader the second time around. Communication was also predicted by the composite language measure and by TEC. The levels of socioemotional comprehension and communicative uses of language and interaction do not necessarily come together but the correlation in the present data appears intuitively plausible. As the communication measure is novel and the measure for socioemotional comprehension consisted of only one test, future studies will have to further investigate the relation between these two areas.

## Conclusion and future directions

As the interventions did not yield results, we have to conclude either that the interventions were not implemented in the right manner, that they were too short, that the groups were too heterogeneous to compare, or that the pedagogical methods in use in preschools are less important for children’s outcomes than what might be expected. Having a high overall quality might be good enough in order for children to embark on their developmental trajectories in the best way they can.

Summing up the discussion on background variables, we can see that SES is an important component even in the typically higher-SES Swedish preschool context. Children with similar backgrounds also tend to live in close proximity to one another and thus attend the same preschools. This entails an obvious risk/opportunity for these children also remaining in the same SES environment. For the lower-SES children this is a critical condition threatening to influence the rest of their lives in a negative way [26, 27]. Although a political issue on the whole, pedagogical practices in Swedish preschools, which reach almost all children from an early age, could well be the best way forward to even out the differences associated with SES. To succeed in this, the pedagogical practices as such need be closely scrutinized with regards to their efficiency and impact. This study was one of the first attempts within the Swedish preschool context to accomplish this, and the lack of conclusive results can be used as a foundation for future attempts.

## Additional files

Additional file 1: SEMLA. Observation protocols. Observation protocols featuring the seven components and the processes of the group and individual children. (DOCX 72 kb)

Additional file 2: DIL. Intervention protocol. DIL. Instructions to teachers for how to implement the digital learning paradigm for Magical Garden and body-and-mind exercises. (DOCX 594 kb)

Additional file 3: All Univariate results and all Multivariate results. All tests for univariate and multivariate regressions. (PDF 53 kb)

Additional file 4: Supplementary ERP data. Supplementary ERP grand average plots for all head electrodes, HEOG and VEOG. 1. All pre intervention 2. All post intervention 3. Control pre 4. Control post 5. DIL pre 6. DIL post 7. SEMLA pre 8. SEMLA post. (XLSX 15 kb)

## Data Availability

The datasets generated and analyzed during the current study are not publicly available due to personal integrity related to our ethics approval but parts of the data (on group level) could be made available from the corresponding author on reasonable request. We are happy to provide openly accessible materials as well as information on how we have proceeded in test management, mobile EEG laboratory set-up, and translation of various materials to Swedish.

## Abbreviations

ADOS: Autism Diagnostic Observation Schedule
ANOVA: Analysis of variance
BRUK: Bedömning, Reflektion, Utveckling, Kvalitet (Assessment, Reflection, Development, Quality)
CMS/DRL: Common Mode Sense active electrode/Driven Right Leg passive electrode
CONSORT: Consolidated Standards of Reporting Trials
DCCS: Dimensional Change Card Sort task
DIL: Individual digital implemented attention and math training paradigm
ECEC: Early Childhood Education and Care provisions
ECERS-3: Early Childhood Environmental Rating Scale, third edition
EEG: Electroencephalography
EF: Executive functions
ERP: Event-related potential brain response
FLP: Family Language Problems
HSKT: The Head-Shoulder-Knees-Toes task
L2: Language two (second language)
MANOVA: Multivariate analysis of variance
MG: Magical Garden
OECD: Organisation for Economic Co-operation and Development
PPVT: The Peabody Picture Vocabulary Test
RCT: Randomised controlled trial
RQ: Research Question
SCDI: Swedish Communicative Development Inventory
SD: Standard Deviation
SDQ: Strength and Difficulty Questionnaire
SEMLA: Socioemotional and Material Learning group paradigm
SES: Socioeconomic status
STEAM: Science, Technology, Engineering, Art and Mathematics
TA: Teachable Agent
TEC: Test of Emotion Comprehension

## Footnotes

1 The intended activities can be found in the documentation formulary (see Additional file 1).

2 The Magical Garden is developed in cooperation between Lund University and Stanford University, see [63]

3 Brain Development Lab at Oregon University, see [66]

4 The activities are described in detail in the manual Body and Mind Exercises (see Additional file 2).

5 See Gerholm et al. [48] for a table overview of hypotheses, analyses, etc.

6 This short notice was needed for practical reasons as many children move or begin preschool even in the middle of semesters and we wanted to only approach families actually at the preschools during the intervention period. Some preschools further gave short notice of participation due to staff situation or other factors beyond our control.

7 See however [88–91] for a critical discussion on the validity of ECERS and Garvis et al. [92] for a discussion on the need of cultural adaptation of the instrument.

8 The seven components consist of: a relational ethics; content and problem-focussed learning derived from an overarching problem of concern; socioemotional and material learning; inclusion, participation and self-management; collaborative and individualized scaffolded learning; aesthetic and multimodal investigations; pedagogical documentation practices as tools for learning [50].

9 A 10-graded scale based on the basis of both parents’ annual income (3 levels were used, 1: 0–200,000 SEK; 2: 200,001-500,000; and, 3: 500,001>) and their educational level (4 levels were used, 1: elementary school only; 2: upper secondary school; 3: vocational education; and, 4: college/university). See Gerholm et al. [48] for further details and explication of calculations used.

10 Comparing SES between countries is hard as the rating is relative. The general low SES within the Swedish or Scandinavian context can be expected to be above the general low SES of, for example, U.S. where poverty is quite wide-spread and have a much lower “lowest” degree, as 83% of the adult Swedes has high school education or more and relative minor income differences compared to most other OECD countries [134].
