# Supplementary material for: A randomized controlled trial to examine the effect of two teaching methods on preschool children’s language and communication, executive functions, socioemotional comprehension, and early math skills
Source: BMC Psychol. 2019 Sep 5;7:59. doi: 10.1186/s40359-019-0325-9 (PMC6729003; doi:10.1186/s40359-019-0325-9)
Supplement: Supplementary file 1 — SEMLA. Observation protocols. Observation protocols featuring the seven components and the processes of the group and individual children. (DOCX 72 kb) [file 40359_2019_325_MOESM1_ESM.docx]

**SEMLA (Socio-emotional and material learning)**

**Observation protocols featuring the seven components and the processes of the group and individual children**

The seven components: (i) a relational ethics, (ii) content and problem-focused learning, (iii) socio-emotional and material learning-environment, (iv) inclusion, participation and self-management, (v) collaborative & individualized scaffolded learning, (vi) aesthetic & multimodal investigations, (vii) pedagogical documentation practices as tools for learning. The observation protocol is constructed for the educators to become aware of what is ideally supposed to take place in the teaching/learning practices of SEMLA simultaneously in relation to the group of children (ideally 5-10 children at the time with 1-2 educators). Educators are instructed to pay attention to a handful of these at the time while either observing another educator or reflect on their own teaching during and after a learning/teaching session. The last part of the protocol is constructed as a summary and reflection of what needs to be considered and planned for the upcoming learning session. Note that the protocol poses similar questions to answer for the group and the individual children respectively. This is due to the fact that some educators tend to favor teaching in relation to a group without targeting individual children while doing so, while others tend to focus mostly on singular children while having problems supporting the group-learning. The idea is for the educators to eventually find a balance in how they address and scaffold the group of learners and the individual children in the group. They are also instructed to pick the below questions that they find more important to pay extra attention to, reformulate them if necessary and add new questions to pay attention to depending on what children are present and depending on the learning contents that are in focus and what materials that are used. If early math is a content and various math tools are used, targeted questions can be formulated in order to promote better observations.

**
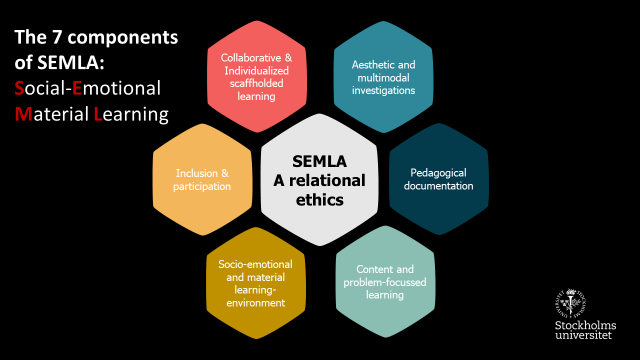
**

| **Date & place/room:** | **The Group:** the names of the participating children | **Present educators:** | **Child 1** | **Child 2** | **Child 3** |
| --- | --- | --- | --- | --- | --- |
| - **Learning contents and the learning environment** - What happens in the group/**what is going on**? - Towards what is the **collective** **attention** steered? - What **words or concepts** are used often and how?   What is **the driving force of the group process**? (An idea, the educator’s scaffolding, a material, etc.?)   - How does **the environment, the room inside and outside affect** the attention and process of investigation of the group/smaller groups?   How does **specific materials** affect the child’s investigations/process? |  | - **Learning contents and the learning environment**   What **does the child do**?  Towards what does the child steer her/his **attention**?   - What **words or concepts** are used often and how? - What **is the individual child’s perspective/take on a matter/phenomenon** – perhaps in relation to who the child is, what it already knows, what it can already do?   How does the **environment, the room inside and outside affect** the attention and process of investigation of the individual child?  How does **specific materials** affect the child’s investigations/process? |  |  |  |
| **Scaffolding, feedback, meta-reflection**   - What does the **educator say** **or do** that has effects on what the children do as a larger or smaller group?   In what situations is opportunity given for children in the group **to pose questions to each other**?  What strategies does the educator use to make the children **reflect about their own thinking?**  In what situations do a child typically support/scaffold or encourage or give **feed-back to another child to promote group-learning**?  When, for what and how does the **educator give the group feedback** or encouragement?  In what situations is opportunity given for a pair or smaller group of children **to prepare for and demonstrate their ideas**, solutions to a problem etc.  When and how are **new words and concepts** introduced?  In what situations are opportunity given **for conceptual development**? |  | **Scaffolding, feedback, meta-reflection**   - What does the **educator say or do** that has effects on what an individual child does?   In what situations is the individual child given opportunity **pose questions to other children or the educator**?  In what situations is opportunity given to each of the **children to speak up/out** to articulate their thinking/theories/ideas/ reflections/emotions?  In what situations and how is the individual child encouraged to **think in new or other ways**?  In what situations and how is the individual child encouraged to **use new words or concepts and engage in conceptual constructions and development**?  In what situations and how is the individual child **encouraged to think about her/his own thinking**?  In what situations is opportunity given for an individual child **to prepare for and demonstrate their ideas**, solutions to a problems to other children?  In what situations is the individual child given the opportunity **to repeat and practice a skill**? |  |  |  |
| - **Affect and emotions in learning**   In what situations is the group of children encouraged to *together* **explore their fantasies, involve their perceptual affects and emotional expressions** as part of the learning process?   - In what situations is it possible to observe the group of children in **moments and spaces of intense focused attention and/or perceptual affective intensity and/or emotional density**?   In what situations is it possible to observer – on the group level – strong **engagement in others’ situations/emotions and/or empathetic expressions**?  When and how do the children in the group **encourage each other emotionally**?  When and how does **the educator** address and support the group emotionally and/or empathetically?   - **What emotional expressions or articulations** appears to affect the group-process and how? |  | - **Affect and emotions in learning**   In what situations is the individual child encouraged to **explore her/his fantasies, involve her/his perceptual affects and emotional expressions** as part of the learning process?  **What emotions seem to function as a driving force** for the individual child’s learning? How can this be observed?  When and how, and with what effect, does **the educator emotionally encourage/scaffold** the individual child?  In what situations does the individual child **get excited in relation to its own learning?**  In what situations and/or by ways of **what emotions is the child’s focused attention** engaged?   - **What emotional expressions or articulations (from others or her/himself)** appears to affect the individual child’s learning and how? |  |  |  |
| **Ethics and participation**   - **When and how are negotiations (or elections)** around contents and activities conducted with the children as a group?   Are the children given **opportunities to reflect together** on different issues to come up with new solutions or suggestions to the larger groups’ learning-process?  When and how **are different choices of learning-focus or activities** made possible and presented to the whole group?  **Whose will or perspective is strongest** in the different choices made and directions taken in the group-learning process? (It should not always be the educators but not always the children’s will or perspectives either.)  **When and how is difference** in terms of the difference between children’s ideas or solutions made productive for what is going on in the group-learning process?  In what ways are the children understood to articulate their knowledge, experience and emotive engagements, **and thereby understood to (at least in part) be able to claim ”ownership” of their learning-processes** ***as a learning group?*** |  | **Ethics and participation**   - To what extent is the **individual child’s articulations of ideas, strategies, experiences and emotive engagements actively used** as part of the learning-process? - In what situations and how can the **individual child be seen to affect the learning** of another child, or the thinking and choices of the adult educator?   When and how does the educator **encourage the individual child’s participation** in her/his individual learning process?  When and how does **the individual child’s articulations and perspectives made productive** in the larger group-learning process?  In what situations (and why) does the individual child chose to **depart from the group to engage in individual activities** connected to the group learning process, or entirely other things not connected to the group-learning?  In what ways is the individual child understood to articulate her/his knowledge, experience and emotive engagements, **and thereby understood to claim ”ownership” of her/his own individual learning process**? |  |  |  |
| **Aesthetic, ethical and multi-modal learning & pedagogical documentation**  When, how and why are **new materials and artefacts (or places to learn**) introduced to the group-learning process?  **How and with what effect are new materials used to translate and transpose a problem from one material to another**; i.e. stimulating new perspectives and cognitive abilities?  When and how are the children encouraged to make use of **the body and other bodily perceptions and sensations** to learn?  When, how and why are specific learning-processes in the learning group **documented**?  How is the **documentation later used as a tool** in the group-learning process?  When, how and why are the children **encouraged to document each** other during the group-learning process?  **What strategies of ethics** are presented and used as part of the group-learning process? (i.e. stop-hand, and specific words, children selecting their own images and films etc.) |  | **Aesthetic, ethical and multi-modal learning & pedagogical documentation**  What **materials and artefacts affect the individual child’s attention and concentration** on the task?  What **materials evoke in the individual child positive emotions and joy** of learning?  **How and with what effect are new materials used to translate and transpose a problem from one material to another** in the individual child’s learning?  When and how is the individual child **given the opportunity to use its body and other bodily perceptions** and sensations to learn?  What **abilities of the individual child** can be observed to develop in relation to specific materials, spaces or activities?  What **abilities of the individual child can be observed that need to be further scaffolded** or challenged in order to develop?  When, how and why is the individual child **encouraged to document? And how is documentation** used to scaffold its learning? |  |  |  |

**POST-REFLECTIONS to inform further SEMLA learning-processes based on an analysis of the above documentation**

| **Date & place/room:** | **The Group:** the names of the participating children | **Present educators:** | **Child 1** | **Child 2** | **Child 3** |
| --- | --- | --- | --- | --- | --- |
| - What is the **focus of the larger group’s attention and interes**t around the phenomena or over-arching problem now studied?   **How do we know this?** What makes me/us think that it is that tool, material, change of spaces, idea or those concepts that we should continue working with? Are the children in any way part of the choices made? (important in order to evaluate our decisions)  What ***Activities; Materials/tools, Spaces of learning, Ideas and Central Concepts*** seem most relevant to continue working with for the group?  How have we used the **documentation done by children and ourselves to know more about how to proceed**?   - **What are our intentions for further learning?** Which are the potential activities that become available for the children if we proceed in this fashion?   Are there **alternative ways/ strategies** to proceed which we can activate if necessary? |  | - **What is the foucs of the individual child’s attention and interest** around the phenomena or over-arching problem now studied?   **How do we know this?** What makes me believe this is the fact? Has the child participated in articulating and evaluating its’ own interest?  In what ways should the **individual child’s abilities and knowledge- production** be addressed and supported/ scaffolded?  What ***Activities; Materials/tools, Spaces of learning, Ideas and Central Concepts*** seem most relevant to continue working with in relation to the individual child?  How have we and can we **use the** **documentation** produced by the child or ourselves to support the child’s learning?  Are there **alternative ways/strategies** to proceed to encourage the child’s learning which can be activated? |  |  |  |
