# Supplementary material for: A randomized controlled trial to examine the effect of two teaching methods on preschool children’s language and communication, executive functions, socioemotional comprehension, and early math skills
Source: BMC Psychol. 2019 Sep 5;7:59. doi: 10.1186/s40359-019-0325-9 (PMC6729003; doi:10.1186/s40359-019-0325-9)
Supplement: Supplementary file 2 — DIL. Intervention protocol. DIL. Instructions to teachers for how to implement the digital learning paradigm for Magical Garden and body-and-mind exercises. (DOCX 594 kb) [file 40359_2019_325_MOESM2_ESM.docx]

## Additional file 2: Intervention DIL (individual digital implemented attention and math training paradigm)

## DIL-hour – 6 week plan

Start each DIL-hour by gathering all children in a circle on the floor. Focus the attention in the group by instructing all children to take three deep breaths. Say “Now it is DIL-hour, that means we will work with our attention and do what we can to be focused”. In addition to the actual exercises, some time each session is spent reflecting how playing the MG is coming along. “What is fun? How are the teachable agents learning? What can you do if it feels boring or repetitious?”

| WEEK 1 |  | Notes |
| --- | --- | --- |
| Day 1 | Introduce DIL for Body and Mind using the Power Point  Put up and introduce the CALENDAR  Introduce Magical Garden  Go through the DIL-room rules  PLAY Magical Garden(MG) |  |
| Day 2 | Introduction with the CALENDAR  Put up and introduce the ATTENTION POSTER  Talk about the learning body and about the importance of the brain  PLAY MG |  |
| Day 3 | Introduction with the CALENDAR  Put up and introduce the BIRD BREATH POSTER  Connect to the ATTENTION POSTER  PLAY MG |  |
| Day 4 | Introduction with the CALENDAR  Use the strategies  Introduce the TEACHABLE AGENTS/Learning Buddies  Introduce BALANCING THE TEACHABLE AGENTS PLAY MG |  |
| Day 5 | Introduction with the CALENDAR  Exercise BALANCING THE TEACHABLE AGENTS  Use the strategies PLAY MG |  |
| WEEK 2 |  |  |
| Day 1 | Introduction with the CALENDAR  Repeat BIRD BREATH and ATTENTION POSTER  Put up and introduce the OH WELL POSTER  PLAY MG |  |
| Day 2 | Introduction with the CALENDAR  Use the strategies  BALANSING WITH TEACHABLE AGENTS PLAY MG |  |
| Day 3 | Introduction with the CALENDAR  Use the strategies  BALLOON BLOWING/focus breathing &attention  PLAY MG |  |
| Day 4 | Introduction with the CALENDAR  Use the strategies  BALANSING WITH TEACHABLE AGENTS PLAY MG |  |
| Day 5 | Introduction with the CALENDAR  Use the strategies  BALLOONBLOWING  PLAY MG |  |
| WEEK 3 |  |  |
| Day 1 | Introduction with the CALENDAR  Use the strategies  Introduce PIN WHEEL  PLAY MG |  |
| Day 2 | Introduction with the CALENDAR  Use the strategies  Introduce BELLY BREATHING WITH TEACHABLE AGENT  PLAY MG |  |
| Day 3 | Introduction with the CALENDAR  Use the strategies  BELLY BREATHING WITH TEACHABLE AGENT  PLAY MG |  |
| Day 4 | Introduction with the CALENDAR  Use the strategies  Introduce PARTY PIN WHEEL  PLAY MG |  |
| Day 5 | Introduction with the CALENDAR  Use strategies  Exercise of your choice from week 1-3  PLAY MG |  |
| WEEK 4 |  |  |
| Day 1 | Introduction with the CALENDAR  Use strategies  Introduce CRANE  PLAY MG |  |
| Day 2 | Introduction with the CALENDAR  Use strategies  CRANE  PLAY MG |  |
| Day 3 | Introduction with the CALENDAR  Use strategies  Introduce BALANCE BALLOON  PLAY MG |  |
| Day 4 | Introduction with the CALENDAR  Use strategies  BALANCE BALLOON  PLAY MG |  |
| Day 5 | Introduction with the CALENDAR  Use strategies  Exercise of your choice from week 1-3  PLAY MG |  |
| WEEK 5 |  |  |
| Day 1 | Introduction with the CALENDAR  Use strategies  Introduce EWER  PLAY MG |  |
| Day 2 | Introduction with the CALENDAR  Use strategies  EWER  PLAY MG |  |
| Day 3 | Introduction with the CALENDAR  Use strategies  Introduce TEACHABLE AGENT THROW  PLAY MG |  |
| Day 4 | Introduction with the CALENDAR  Use strategies  TEACHABLE AGENT THROW  PLAY MG |  |
| Day 5 | Introduction with the CALENDAR  Use strategies  Exercise of your choice from week 1-4  PLAY MG |  |
| WEEK 6 |  |  |
| Day 1 | Introduction with the CALENDAR  Use strategies  BALANCING THE TEACHABLE AGENTS  PLAY MG |  |
| Day 2 | Introduction with the CALENDAR  Use strategies  BELLY BREATHING WITH TEACHABLE AGENTS  PLAY MG |  |
| Day 3 | Introduction with the CALENDAR  Use strategies  BALANCE BALLOON  PLAY MG |  |
| Day 4 | Introduction with the CALENDAR  Use strategies  PLAY MG  PARTY  Choose the exercise that children appreciated the most |  |
| Day 5 | Introduction with the CALENDAR  Use strategies  PLAY MG  PARTY  Choose the exercise that children appreciated the most |  |

**Attachment 2: Magical Garden (MG)– instruction**

Magical Garden is a game that should be played individually every day during a 6-week period. Researchers announce the game's creators (researchers in Lund) each child's first name, birth year and month. The researchers in Lund then create individual logins for each child and some extra login for children who are not allowed to join the study. Children can log into their Magical Garden on their own.

This cheat sheet can be set up in the DIL room or be easily accessible.

**Cheat sheet DIL**

- Kids play on each their iPad with headphones on.
- To play, the iPad must be connected to the Internet.
- The online sign-in is done automatically via_______________________________.
- All children have an individual account on the MG. It is important that children only play on their own accounts, not on each others!
- The preschool staff helps the child to log in to MG and play for about 30 minutes.

**Login Magical Garden (MG):**

Click on the Safari icon on your desktop.

♣ Alt. 1: Most likely, you will come directly to the group _____________ in MG. Click the child's name. The child can now start playing.

♣ Alt. 2: If you do not enter directly into MG, type <http://magicalgarden.ida.liu.se>. Click on ____________________________. Click the child's name. The child can now start playing.

**Advice Magical Garden (MG)**

- The purpose of the game is to solve mathematical problems to get water drops to water in order to grow the garden.
- Each time the child starts playing, s/he can choose a new Teachable Agent/Learning Buddie.
- They can also change the color of the Teachable Agent. (One can change the Teachable Agent and color of it during a game round, but we recommend that the child doesn't).
- It is possible to go to the garden at any time.
- One can water the garden only when there is water in the water ewer.
- When the child has come a bit in play, the opportunity to "arrange a party" turns up. We recommend that children wait a week or two before entering the party world. To enter the party world, click on the brown arrow with colorful festoons.

**Troubleshooting**

If the connection does not work. Try to sign in to________________________________.

If the MG program hangs (lags and chews at 0%) it may be because there are other programs open behind. To close them, do the following:

- Double-click the round button at one end of the iPad.
- Close all applications by swiping them up, one at a time.
- If the MG sounds strange or in other ways behave strangely: log out and reboot again.

**Attachment 3: Body & mind exercises**


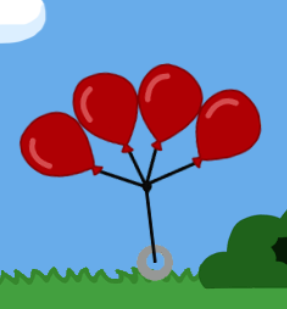

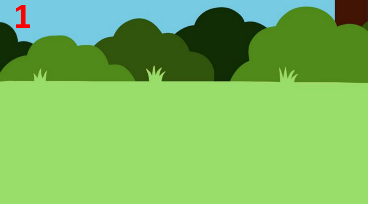

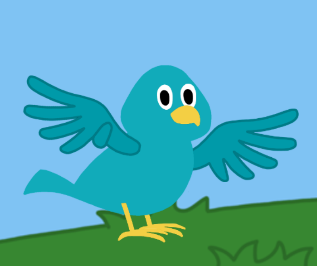

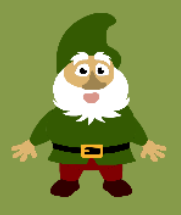

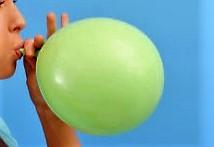

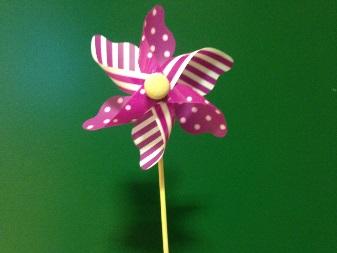

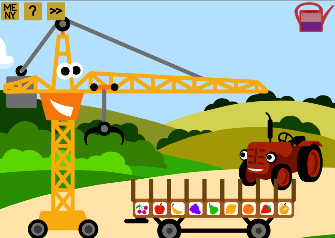

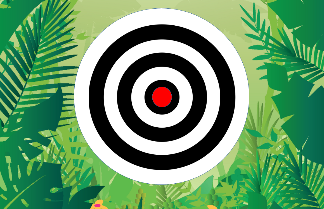

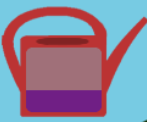


**Body & Mind**

**Exercises**

Body& Mind exercises

[Calendar 9](#_Toc2930153)

[Attention Poster 10](#_Toc2930154)

[Bird Breath 12](#_Toc2930155)

[Oh Well, but I can… 13](#_Toc2930156)

[Balancing with Learning Buddies 14](#_Toc2930157)

[Balloon Breathing 16](#_Toc2930158)

[Party Pinwheel 18](#_Toc2930159)

[Belly Breathing with Learning Buddies 20](#_Toc2930160)

[The Carne 21](#_Toc2930161)

[The Ewer 23](#_Toc2930162)

[Learning Buddie Toss 25](#_Toc2930163)

[Balloon balancing 27](#_Toc2930164)

Acknowledgement:

These Body & Mind exercises are based on the Brain Train materials developed at the Brain Development Lab at Oregon University, see:<https://bdl.uoregon.edu/>. For reference see: Neville, H.J., Stevens, C., Pakulak, E.**,** Bell, T., Fanning, J., Klein, S., & Isbell, E. (2013). Family-based training program improves brain function, cognition and behavior in lower socioeconomic status preschoolers. Proceedings of the National Academy of Sciences, 110(29), 12138-12143.

The original Brain Train exercises have been adapted by Susanne Kjällander and Sofia Frankenberg to fit the Digital Individual Learning for Body& Mind intervention(DIL) that was part of the interventions study [Enhancing Preschool Children’s Attention, Language and Communication Skills: An Interdisciplinary Study of Socio-emotional Learning and Computerized Attention Training](https://www.buv.su.se/english/research/research-projects/early-childhood-education/enhancing-preschool-children-s-attention-language-and-communication-skills-1.209094) led by Prof. Hillevi Lenz Taguchi.

The activities have been adapted to fit the narrative and the design of a digital mathematics game called “Magical Garden” developed by The Educational Technology Group, led by Agneta Gulz at Lund University <https://www.lucs.lu.se/etg/> in cooperation with AAA-lab, led by Daniel Schwartz at Stanford University <http://aaalab.stanford.edu/>. The images are used with their permission.

# Calendar
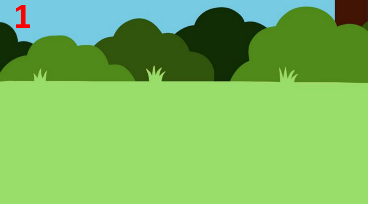


**Brain train inspiration:** Calendar

**Active component:** Attention, self-regulation & meta cognitive awareness

**Material:** Calendar, pictures for the Body & Mind exercises

**Objective:** The calendar is important in order to create a more predictable environment by talking continuously about routines and what is happening during the DIL-hour.

**Introduction:** Start each DIL-hour by looking at the calendar, talk about what you have done during the week/weeks and what you will do today.

**Outline:** 1-5 min

1. Talk about what rules need to be followed in the DIL-room.
2. Ask the children: *Now we are going to talk about our calendar and about what to do each day.*
3. *This calendar shows all the weeks when we will have the DIL-hour in the mornings.*
4. *All these days we will play the Magical Garden and do Body & Mind exercises!*
5. *Raise your hand if you know what we are going to do today!*
6. Show that the calendar has six weeks and let the children say what week you are at now..
7. Put up a picture/es for this days Body & Mind-exercise/es.
8. *Look at this picture! It shows what we are going to do today!*

Feel free to let the children participate in the calendar and tell about what they will do, what they remember from previous DIL- hours and what they expect from today’s DIL-hour.

**Questions for meta reflection:** *How can you know when the next* *Body & Mind exercises will take place?* *How can you know which exercise to do?* *Why is it good to use a calendar?* *How do other people use calendars?*

**Closing and Transition:** Finish the session by letting the children ask 1-2 last questions/comments about the calendar. It is also a good idea to repeat what will happen in the DIL-room during the day/week. Avsluta gärna med att repetera vad som ska hända under dagen/veckan i DIL-rummet.

**Vocabulary:** day, week, calendar

**Variations and improvements:** The children can get calendars of their own to draw or write in. One can show other types of calendars to the children, for example a year calendar, month calendar or your own teacher calendar.

**Pitfalls:** Everything doesn't always turn out as planned and then one needs to re-plan. Tell this to the children with reference to the calendar.

**Notes:**

# Attention Poster
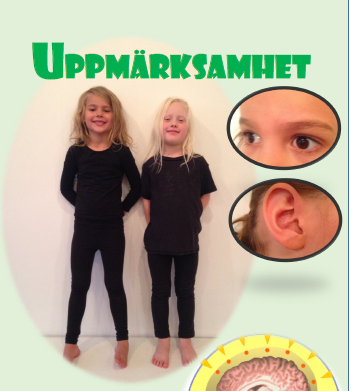


**Brain Train inspiration:** Attention poster

**Active component:** Attention, metacognitive awareness

**Material:** Attention Poster, focus the picture of the brain

**Objective:** Visualize that we all have a learning body and mind (with a brain). Raise awareness about the importance of the brain and how attention is dependant on the body. Describe how the brain looks, where it is located, the size of it (two fists) next to each other), explain that the brain controls everything we do and that the brain tells us what to do!

**Introduction:** Say: Today we are going to talk about the body and mind and about how the body and mind functions and learns.

**Sequencing:** 5-10 min

1. *Now we are going to talk about the importance of the brain when we are thinking and learning things. The brain does many things even if we are not thinking about it. But if we know a bit more about how the brain works we and use it even better. That might be a good thing when we learn how to tie our shoe laces, ride the bike, read by ourselves or perhaps bake some buns. It is also good when we are playing the Magical Garden.*
2. *Who can wiggle their little finger? Your ears heard me say it and you were telling your brain to do it. What connects your eyes, ears, toes and mouth? YOUR BRAIN!*
3. *And who controls your brain? Who tells your eyes to watch, and your ears to listen? YOU!*
4. Show the brain on the poster. *Feel your head: you are feeling your skull- it is the protective skeleton that surrounds your entire soft brain. Your brain is soft and it is very important that you treat it carefully.*
5. *If you put together your fits like this- then you will see approximately how big your brain is.*
6. Look at the picture of the brain again. *What do we do with our brains?* Perhaps you need to give some clues first, but as soon as the children have started to think about the subject, they will probably come with their own ideas. Personal examples are great here and will engage the children.
7. *Your brain is very important in order for you to think, but your eyes, your ears and your body are also important.* Point to your body, your eyes and to your ears on the poster and tell the children that it is themselves that chose to watch and listen, that they themselves chose how to move their bodies or hold their bodies still.
8. It is difficult but important to explain that all information goes via their brain to their different body parts and that every child themselves can choose what he/she focuses their attention on.

**Questions for metacognition:** *What does your brain do? How do you remember things? How do you know your brain is thinking?*

**Closing and transition:** Before the children lose interest you can tell them that you would like to hear one or two ideas for the day. Take a minute to feed back what the purpose of the brain is.

**Vocabulary:** brain, attention, thinking

**Variations and improvements:** Teachers will be referring to and talk about the Attention poster multiple times during the DIL-intervention.

**Pitfalls:**

**Notes:**

# Bird Breath
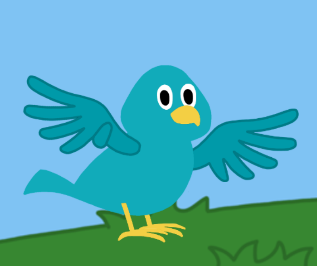


**Brain train inspiration:** Bird Breath

**Active component:** Attention, self-regulation, metacognitive awareness

**Materials**: Bird Breath Poster

**Objective:** Raise awareness about breathing and how breathing can be used consciously for self-regulation and focused attention.

**Sequencing:** 5 min

**Introduction***: Say: Today we are going to learn the Bird Breath and how that can help us in different ways.* *Introduce the exercise by taking a deep breath.* Make an exaggerated movement with the shoulders up towards the ears while taking a breath*.* Breathe out slowly. *Have you thought about how it feels when you breathe? Try! Listen to me:* (breathe in and out) *Breath in and at the same time pull your shoulders like this* (show). *Breathe out* (let your shoulders down) *Try one more time*.

1. *Do you sometimes take a deep breath? How does that feel?*
2. Tell the children that you breathe in order to get oxygen. In the air there is oxygen. Oxygen is needed in order for the body to function. Oxygen is also needed for the body and brain to work.
3. To think about breathing at the same time as you breath can help the brain to calm down and think clearly in different situations. Perhaps especially when something feels hard or difficult. Think about if you spill out your milk or if someone comes and takes your spade. Before you say something, you can think about taking a deep breath and that can help you calm down so that you can think clearly.
4. *When can it be good to take a bird breath?* Let the children come with suggestions.
5. Summarize the discussion with the following: *You can take a deep breath when you are stressed, need to focus or calm down. When we are playing the Magical Garden and it feels difficult or when you want to stop, try taking a Bird Breath!*

**Questions for metacognition:** *How does it feel in the body when you take a deep breath? How does it feel in the body when you are anxious/ nervous/ stressed? How do you breathe then? How does it feel in your body when you are calm/happy/ content? How do you breathe then?*

**Closing and transition:**

**Vocabulary:** calm, breath, focus, stress

**Variations and improvements:**

**Possible pitfalls:**

**Notes:**

# Oh Well, but I can…
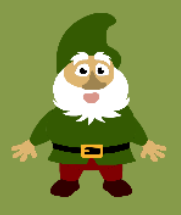


**Brain train inspiration:** Oh Well…

**Active component:** Attention and managing distractions, self-regulation & metacognitive awareness.

**Materials:** Oh Well poster

**Objective:** Introduce ”Oh well, but I can…”. The aim is to give the children a strategy for how they can think differently, put words to and change focus when they feel they are restricted to by being distracted, sad, bored, frustrated…

**Introduction:** *Today we are talking about what we can do if we feel frustrated.*

**Outline:** 5 min

1. *Does anyone know how it feels to be frustrated? Är det någon som vet hur det känns att vara frustrerad?* Talk to the children about what it means, about how it feels when something goes wrong or not as you want it to.
2. *Here is a good thing we can do if we are frustrated: It is called Oh well, but I can…” Let’s practice together. Everyone makes a funny face that shows how it feels when something goes wrong, for example if you have built a high tower that falls down before you are finished. Then we all take a joint big breath, shake your shoulders and say, “Oh well, but I can try one more time!”*

**Questions for metacognition:** *When can you use ”Oh Well but I can…”? När kan du använda ”Oj då, men jag kan…”? Do you think you could help a friend and remind about ”Oh Well but I can”?*

**Closing and transition:**

**Vocabulary:** Attention, Frustration, Distraction

**Variations and improvements:** The exercise can be developed by using two puppets. One puppet plays frustrated or angry and the other tells the first how it feels. The second puppet turns to the children and asks what you can do if you feel frustrated.

**Possible Pitfalls:**

**Notes:**

# Balancing with Learning Buddies
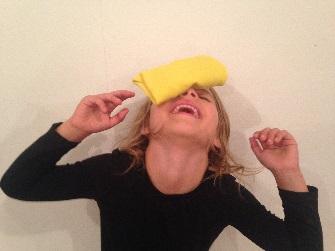


**Brain train inspiration:** Bean Bag Balancing

**Active component:** Attention, self-regulation & metacognitive awareness.

**Materials:** Bean Bags (We call them Learning Buddies together with the children but they represent Teachable Agents theoretically and in the Magical Garden game).

**Objective:** To increase Mind/Body awareness by balance and control. To practice using the strategies Bird Breath and Oh Well, but I can…”

**Introduction:** Tell the children that it is time for a balancing activity. To train balancing is a great way to help children become more aware of their capacity to control their bodies.

**Outline:** 5-10 min

1. Look at the Attention poster. Pay attention to the eyes, body and brain. *Today we are doing a fun exercise in order to learn to control our bodies with our brains, while we are balancing the Learning Buddies*
2. Before you distribute the bean bags you can ask the children to balance on one leg. Show how it looks when you balance on one leg. Wave your arms while you continue to balance the bean bag.
3. Tell that they now will balance with their Learning Buddies (bean bags). Also say that it the bean bags should not be thrown in this activity.  *Today we will feel how it feels to pay attention and focus our bodies. We are going to balance.*
4. Start by letting them stand with both feet on the ground and with the bean bags in their stretched arms. Remind the children that they must take breaths to calm down and to focus throughout the entire activity.
5. *Try to lift one of your legs. Focus your attention by thinking about what you are doing. Don’t forget to breathe.*
6. Ask them to change the leg that they are balancing on. Thereafter, you ask the children to balance in different ways with the bean bags on different parts of the body: on their head, foot etc. You can ask them to lift an arm or a leg at the same time as they are balancing. Remind them that they can say “Oh Well, but I can…” if they drop the bean bag.
7. Increase the difficulty gradually but make sure that everyone succeeds in balancing the bean bag for a while.

**Questions for metacognition:** What would happen if you did not focus on your body? Why did we take a breath before we balanced our Learning Buddies? What part of you helped you to control your body while you were balancing the Learning Buddy?

**Closing and transition:** The exercise Learning Buddy Toss can be used as ending.

**Vocabulary:** Balance, shift, lift, knee, elbow, wrist, shoulder

**Variations and improvements:** Balance the bean bag on your shoulder, elbow, head or knee. Go through the room while you are balancing a bean bag on a part of your body. Bend over and touch the floor while you are balancing the bean bag on a part of your body. Let the children give each other specific praise. Combine with the strategies Bird Breath and “Oh Well, but I can…”

**Possible Pitfalls:** Some children might want to throw the bean bags. If the children find it difficult to balance they can take a break and watch the others for a while. The exercise can be good to do individually or in smaller groups in order to facilitate independent practice, individual interaction and specific feedback.

**Notes:**

# Balloon Breathing
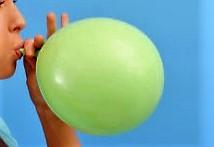


**Brain train inspiration:** Balloon breathing demonstration

**Active component:** Self-regulation & metacognitive awareness

**Materials:** Balloons, Bird Breath Poster

**Objective:** To empower children to be aware of breathing as a tool to manage emotions. Breathe awareness/control, body awareness/control, awareness of when to take a breath.

**Introduction:** Children love balloons and they will naturally be interested and engaged in connecting to their breath and practicing self-regulation.

**Sequence:** 5 min

1. Without saying anything, show the deflated balloon and take an exaggerated breath to blow up the balloon. Then look approving at the balloon and take another big breath in order to blow up the balloon even more. Blow again and show with your arms that the balloon is getting bigger and bigger. *How did I blow up this balloon? Our breath is an invisible power that we cannot see until it is inside the balloon!*  Extend their attention by making the balloon squeak while letting the air out, then tell kids the balloon is going to fly around, but we can only sit and watch – we will not chase the balloon. *How will you control your body so that it does not run around chasing the balloon? Are you going to sit on your hands? Can you control your body in any other way?*
2. After the balloon has flown around in the air give the children lots of specific praise for sitting down. *How did you manage to control yourselves?*
3. Now ask the children to take big breaths together with you while you blow up the balloon. Make a big fuss about the fact that the children are participating in blowing up the balloon together with you. When you let the balloon go and fly around in the air give the children lots of praise when they manage to sit still.
4. Share some examples of when it is good to be able to take a deep breath. *Can you think of some situations when it can be good for you to take a deep breath?* Make sure to explain how breathing calms us down, helps us focus and gives us energy and help our brains to think.

**Questions for metacognition:** *When is it important to take a breath? How can you remember to slow down and take a deep breath? Are there other ways (than to sit and watch a balloon flying around) and focus your breath?*

**Closing and transition:** *Now we will blow up the balloon one last time!*

**Vocabulary:** breathing, breath, breath in, breath out

**Variations and Enhancements:** Use this Body & Mind exercise as an introduction to balloon balancing. The Children can breathe on a cold window in order to see their breath. The children can also see this if it is minus degrees outside in the yard or excursion.

**Possible Pitfalls:** The children may get overly excited over the balloon in the room and disappointed because they are not allowed to play with it. Point at the weekly calendar on the wall and tell them that later that week or another week they can play with the balloon when it is time Balloon Balancing and the Party.

**Notes:**

# Party Pinwheel
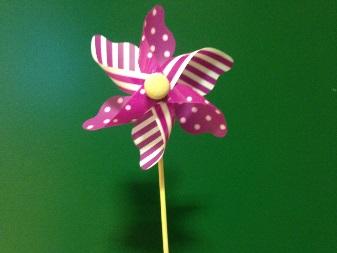


**Brain train inspiration:** Pinwheel breathing

**Active component:** Self-regulation & metacognitive awareness

**Material:** One Pinwheel for each child, Bird Breath Poster

**Objective:** Raise awareness about breathing and to control breathing using your brain, learning about the difference between blowing hard and soft.

**Introduction:** Introduce the Party Pinwheel by mentioning the party in the Magical Garden and reminding about the party by the end of DIL, when six weeks have passed. *But already now we are going to do something with the pinwheels*. Tell the children that they will learn about breathing using the pinwheel. The activity will give the children the opportunity to play with a pinwheel at the same time as they learn about the power of their own breath. *Breathing, like the wind is an invisible power.*

**Sequence:** 10-15 min during circle time.

1. Show the children a pinwheel and say that they will soon get one each. They are now going to use their *brains* in order to control their *bodies* and their breath.
2. Show how you can make the pinwheel turn using your breath. Say it is almost like magic, to be able to make the pinwheel move without touching it. Talk about the importance of breath and the power that each breath means in order for us to live (The human being can live many days without food and without water a couple of days, but without breathing we can only live for a short time). *We can control our breathing even if we normally do not think about it. The body makes us breath by itself. But it can sometimes be good to think about that we breathe.*
3. Show how you can control how the pinwheel spins by blowing hard or soft. *What happens if we blow softly? What happens if we blow hard?* Ask one child to show the others. Who can blow so that the pinwheel spins slowly? (Select one child to demonstrate). Let the children blow one at a time.
4. Distribute one pinwheel to each child and let them explore how it works. Ok, everyone now blow so that the pinwheel spins fast. And then (with a soft voice), ok, who can make it spin slowly? Shift back and forth in order for the children to experience how it is to blow hard and soft. Now everyone can blow fast one last time and then we will finish.
5. Label all attempts to regulate the breath and the blowing. Give specific praise to the children who made the wheel spin slowly. In the exercise, the children will experience how it is to think about and control their breath, by changing between blowing hard and soft. Focus on how one can control the breath.
6. Close the exercise by instructing the children to one last time blow softly while controlling their breath. Say: *There, now we will finish this exercise. Now try one last time and blow softly.*

**Questions for metacognition:**  *What parts of the body helped you to make the pinwheel spin fast AND slow? When does it happen that you breathe fast? When does it happen that you breathe slowly? When would you like to take a deep breath to calm down?*

**Closing and transition:** Close the exercise by saying: *Now is the last chance to blow your pinwheel! Start blowing really slowly and blow more and more so that the pinwheel spins faster and faster.* Collect all pinwheels

**Vocabulary:**

**Variations and improvements:** The children can be instructed to make the wheels turn in the same speed. Let the children challenge each other and blow as softly as possible/ make the pinwheel turn as slowly as possible.

**Possible pitfalls:** Depending on the type of pinwheel it can be more or less difficult to make the wheel spin. Help the children so that it works.

**Notes:**

# Belly Breathing with Learning Buddies
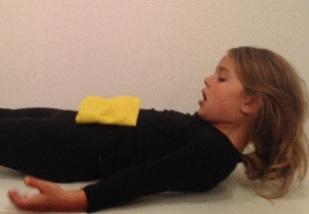


**Brain train inspiration:** Belly Breaths with Stuffed Animals. (We do the exercise individually instead of in pairs).

**Active component:** Attention, self-regulation, metacognitive awareness

**Material:** Bean bag for each child, Bird Breath Poster

**Objective:** To train deep breathing, find an inner calmness, relaxation.

**Introduction:** Today we are going to train relaxation and belly breathing with your Learning Buddies.

**Upplägg:** 7-10 min

1. All children sit down in a circle. Ask: *When can it be good to take a deep breath?* Discuss what you can do to become calm and how you can become focused.
2. Instruct all the children to lay down on their backs and to breathe calmly and to relax. Ask the children to feel how it feels in the body and belly when they breathe deeply. Say: *Now we are going to play with our Learning Buddies in order to learn more about how the breath works. Lay down with your arms along your sides. Hold your Learning Buddy in one hand. Close your eyes. Listen to your breath… now place the Learning Buddy on your belly. Breathe calmly. Now try to take a deep breath so that the air goes down all the way to your belly. What happens to your Learning Buddy? Can you breathe so that it goes up? What happens when you breathe out?* Let the children try for a while.

**Questions for metacognition:** How does it feel in your body when you relax and breathe deeply? What parts of the body controls breathing? When can it be good to train your breathing? How can we know when it is good to take a deep breath? How can we know if someone else needs to take a deep breath to calm down?

**Closing and Transition:** Ask all children to sit up again and to reflect together about how it felt. *Now place your hand on your belly. Take a deep breath, can you feel that the belly goes up when you breathe in?*

**Vocabulary:** breathing, lungs, relaxation

**Variations and improvements:**

**Possible Pitfalls:** The children may want to play with their Learning Buddies instead or interact with one another. Use specific positive feedback in order to direct the children throughout the activity.

**Notes:**

# The Carne
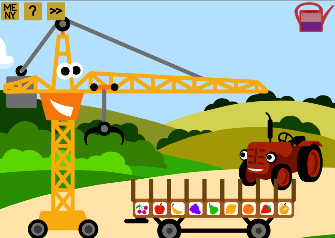


**Brain train inspiration:** Tightrope Walk

**Active component:** Attention, self-regulation / metacognitive awareness

**Materials:** small plastic fruits, balls or other small objects, spoons, tape

**Objective:** Raise body & mind awareness, practice keeping visual focus, breathing calmly, controlling your body and focusing eyes.

**Introduction:** Inform the children in advance that they are going to practice being Cranes and to transport fruits on a small road in the harbour. The activity gives the children a task to focus on while they coordinate their brain and body.

**Sequencing:** 10 min

1. Look at the Attention poster focusing specifically the eyes and body. Ask the children if they have ever seen anyone line dance. Tell them that they will be practicing their own balance by walking on a narrow road in the harbour where the crane is standing in the Magical Garden. To balance on the narrow road. *If you step of the narrow road the fruit will fall into the water.*
2. Show the children how you have taped a path in the room and how they are supposed to follow the path by placing their big toe on the tape in each step they take. We are going to practice “Oh Well, but I can…” while we play a game. *We have all this fruit that we must transport to the right place. Everyone is going to be a crane. You will get one spoon each and you will carefully transport the fruit on it. You will go along the narrow path without dropping the fruit.* The first time you play the game all the children can do it at the same time, but late the children can do the exercise individually when they need to take a short break between playing the Magical Garden.
3. Show the children how they can be cranes, move the spoon up and down a couple of times. Then drop the fruit intentionally on the floor. Look at the children and say: *Oh, I feel upset because I dropped the fruit. What should I do now?* Give the children a clue: *Oh well, but I can...try again. Let’s practice!* Take a deep breath. *Oh Well, but I can try again!* Show how you can go slowly with strong focus. *Can you see how I am doing? What do you see me doing?* (take a deep breath, focus your eyes, go slowly, try again).
4. Encourage the children to focus the gaze on the narrow path while they are walking in the line. Remind the children that they must think about where their bodies are and concentrate on balancing by keeping their body control while they are walking. *Are you ready to transport your fruits to the right place now?* Refer to the Party pinwheel and how important it is to take a deep breath before you concentrate on something. Let the children stand by the starting point of the road. *What do you need to do in order to calm down your body before you start?* Tell the child to drop the fruit intentionally somewhere along the narrow path in order to train ”Oh well, but I can…”
5. Now it is time to do the Crane exercise with distractions. The teacher walks on the taped narrow path with fruit on the spoon. One child is instructed to distract the teacher in some way. The child is not allowed to touch or throw things at the pedagogue in any way, just show, call and make different sounds.
6. The teacher shows: focus, deep breathing, turn away the body from the distraction, getting distracted but saying: *I feel distracted because the Magician is calling that there is a party, so I must turn away (this refers to content in the Magical Garden).* While the teacher is walking on the narrow line she/he can let herself/himself be distracted and drop the fruit from the spoon. *“Oh well, but I can…try again”.*
7. Give a lot of specific praise to the children when they succeed in ignoring the distractions and holding their brains focused on walking on the narrow path and transporting the fruit on the spoon.
8. Let all children walk on the narrow path while other children one by one do different distractions.
9. Sit in front of the child that is about to walk on the narrow path, look the child in the eyes and make her/him aware of body control and focus before they start walking: *Take a deep breath, take slow steps, keep your eyes and feet on the narrow path!*

**Questions for metacognition:** What was challenging in this exercise? How could you keep calm even if you dropped the fruit? What was the first thing you did to calm down? How could you keep your balance when you walked on the narrow path?

**Closing and transition:** Inform the children that all children may go more than once on the narrow path. Encourage the children’s attempts to focused attention, even if they drop the fruit. Praise them specifically for how focused they were before, and for how they focused while balancing the fruit on the spoon.

**Vocabulary:** balance, patience, caution, balance, effort, persistence

**Variations and improvements:** For increased difficulty, the path can be made uneven by tracing the tejp over pillows and boxes that the children walk over. Use different objects (different sizes and materials) like fruit. In order to challenge the child he/she can get a spoon in each hand and in that way need to transport two fruits at the same time. They can also be given a smaller spoon.

**Possible pitfalls:** Children can experience feelings of failure if they do not manage the task. In that case remind them to take a deep breath and say: *”Oh well, but I can…”* Some children may want to do the exercise as a race and can need to be reminded that the exercise is about balancing. To keep the fruit on the spoon and keep calm is what is most important.

**Notes:**

#
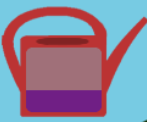
The Ewer

**Brain train inspiration:** Dr Distraction

**Active component:** deal with distractions, attention, self-regulation/metacognitive awareness

**Material:** Watering ewer with or without water, plant (it can be a real one or an image of a plant), learning buddies, Attention-poster, Bird breath-poster, Oops, but I can-poster

**Objective:** To focus on dealing with distractions

**Introduction:** Please inform pupils a while before the exercises that they shall water plants in the magical garden, but that they will also be distracted by different things at the same time

**Outline:** 10 min

1. Tell the kids that their brain can get confused and distracted if you try to get it to do too many things at the same time.

2. For example, when watering a flower, it is important to focus your eyes and body to that the water shall come on the flower.

3. Give the example of how the learning buddy will try to water a flower in the magical garden and get distracted when a robot rolls past, a butterfly flies around or the wizard comes and cries out that it's a party.

4. Show with the water ewer how important it is to focus on watering in the right place even if there are many distracting activities around. Show what happens if you lose focus on the water ewer and look in another direction – then you will water next to the flower instead of on the flower.

5. Say: *Imagine that you are holding a water ewer and that you gently water a flower when a distraction pops up. The bumblebee sits down on the ear, Camilla the chameleon will lick your foot etc...*

6. Say: *Raise your hand if you water flowers sometimes! Your brain needs to focus on watering the flower, if you look away you will spill the water. Sometimes we get distracted, like when a friend cries out hello while we water. Pretend now that you are holding a water ewer and that you are watering a flower.*

7. Then you lead away everyone's attention: *Look, now the robot comes in! Oops, we spilled all the water! DISTRACTION!*

8. All children bring out their learning buddies and one child at a time may try to distract the teacher to water next to the flower. (The child may try, touch the learning buddie in front of the teacher, sound in different ways, but the child may not touch the teacher).

9. Say: *Now we are going to practice to handle distraction and not to be disturbed even though we are distracted. We will practice to focus on what we do.*

10. The educator points to the Attention-poster and on the ´Bird breath-poster and shows how he/she takes a deep breath, stands still and looks with his/her eyes on the water ewer and the flower. *I feel distracted, but I focus, take a deep breath, turn my body away from the distraction and tries not to listen.*

11. One child at a time can then stand with the water ewer at the flower, and the other children can try to distract the watering child with their learning buddies so that the child waters outside.

12. Before each child starts watering, sit down in front of the child, look him/her in the eyes, remind the child to take a deep breath to get body control and to focus before starting to water.

13. When the children get distracted, they can be encouraged to say "Oops, but I can... try again."

| **Questions for meta reflection:** *How does it feel to play Magic Garden with headphones? How does it feel to play without? How does it feel when you get distracted? What do you do when you do not want to be disturbed? What can I do to not be disturbed? What is it that can become distracting in preschool, for example on circle time? How have we just learned that you can manage distractions?* |
| --- |
| **Closing and transition:** Encourage all attempts of focused attention, even if the child allowed themselves to be distracted. Encourage children who distracted their friends and at the same time, regulated their impulses by not touching or throwing something at them. |
| **Vocabulary:** distraction, balance, patience, careful, balanced, effort, persistence |
| **Variations and improvements:** Children need not only use their learning buddies to distract the one that is watering, it can also work with inflated balloons that are bounced around or fly around when the air goes out of them. |

**Notes:**

#
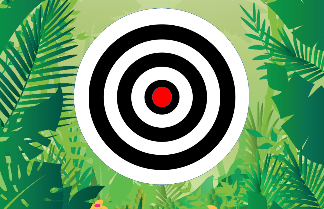
Learning Buddie Toss

**Brain train inspiration:** Bean Bag Target Toss

**Active component:** Attention, self-regulation& meta cognitive awareness.

**Material:** Learning Buddies (the bean bags), target poster, attention poster

**Objective:** Increase focus through visualization and awareness of that each child can control his/her body with his/her brain. The children will focus their eyes on the target, control his/her body, using his/her brain and take a breath to calm down before throwing he bean bag.

**Introduction:**  Tell the children a moment before that they will throw their Learning buddies into the target in the Magical Garden. This to give them a chance to focus their attention beforehand. Preferably, you can do this exercise when children put aside their Learning buddies after using them during DIL-hour.

**Outline:** 5-10 min

1. Look at the attention-poster and talk about how to be attentive with both eyes, body and brain. Explain that they will learn to control their bodies with their brains when they throw their Learning buddies in the goal, i.e., on target in the Magic Garden. *Today we'll know how it feels when we use our brains to pay attention and how it feels when we focus our eyes and our bodies.*
2. Show a moderate distance, a light under hand swing, then close your eyes and talk about how you visualize yourself throwing the Learning buddie BEFORE you throw it. *Take a deep breath, focus your eyes on the goal, relax your body and imagine how the Learning buddie hits the target.*
3. Children are sometimes more motivated when they see a friend because they can imagine themselves doing the activity. *Raise your hand if you think you can do this.* Instruct the children to start on their bottom, then stand on their knees and then stand directly above the target and then take a few steps back again to throw the Learning buddy in goal.
4. You can ask questions to the children who are watching so that they can think critically about the practice: *What did the person know in order to hit the target? Did she/he take a deep breath to be calm first?*
5. The exercise can be repeated several times with longer and longer distances. You can continue by asking the question: *Raise your hand if you feel you are ready to calm your body and focus on the goal.* When the child comes, you can cover the goal, look the child in the eyes and remind him/her to focus his/her attention on the task by providing the following advice:

● Take a large bird breath

● Focus your eyes directly at the target

● Imagine the Learning buddy hits the target directly

● Stand balanced on both legs

● Swing your arm straight back and then straight ahead, as if you'd go bowling

1. Ensure that every child is given the chance to succeed, two to three times.
2. Pay attention to all the good effort even if they do not reach the target and give specific praise to each child.

**Questions for meta reflection:** *What would happen if you had thrown the Learning buddy without looking at the target? Why did we take a deep breath before we threw the Learning buddy? What part of you helped to control your body when you threw? Does it feel different than usual when you take a deep breath and relax before you begin throwing?*

**Closing and transition:** Announce that each child gets more than one toss. When each child has made their last toss, you can ask them what helped them throw straight on to the target.

**Vocabulary:** aim, breath, relax, imagine, focus

**Variations and enhancements:** Encourage the kids to try throwing with their left hand if they are right handed and vice versa. Encourage children to throw while closing their eyes on one eye. Vary the distance. Let the children give each other specific praise. *What would happen if you would have thrown the Learning buddy without looking at the target? Why did we take a deep breath before we threw the Learning buddy? What part of your body helped you to control your body when you tossed? Does it feel different when your take a deep breathe and relax before you start to throw?*

**Possible pitfalls:** If there is a long wait, the children can get bored or start playing with the learning buddies. The group can then be divided into smaller groups to enable more independent training, individual interaction and specific feedback.

**Notes:**

#
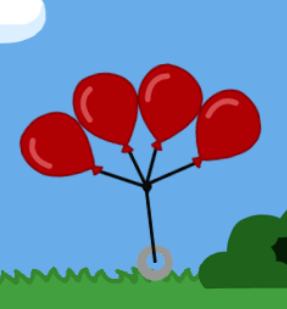
Balloon balancing

| **Brain train inspiration:** Balloon Balancing |
| --- |
| **Active component:** Attention, self-regulation& meta cognitive awareness. |
| **Material:** Balloons |
| **Objective:** To practice body control, both while balancing a balloon and while keeping in mind to keep oneself within the frames of the game (on the carpet or within a demarcation). |
| **Introduction:** *Today we will play with balloons!* |
| **Outline:** 3-5 min |

1. *Let me show you what we should do with the balloons!* Demonstrate how to control the balloon and hold it in the palm of your hand while you sit down, stand up, etc. Show how you constantly keep yourself on the carpet. Show how to take a deep breath and how you relax and focus on the exercise.
2. Raise your hand if you want to try to balance the balloon! Tell the kids where to stand, this can be marked with tape or something else if you don't have a carpet. Tell the children that they should not chase after the balloon if it bounces off.
3. Give the balloon to each child and allow him/her to balance while they are sitting at first. Give them a lot of specific encouragement and let them move the balloon to other body parts. Now try to balance the balloon on the back of your hand, on the elbow, on the foot...
4. Say: *Stand up slowly and maintain control of the balloon in your hand.* When the child is standing up, ask the others: *Did you notice how he/she manages to control the balloon?*
5. Remind the children to focus the eyes on the balloon and move their bodies slowly to maintain control of the balloon. Remind the children to stand still on the carpet. Give immediate specific praise when a child nearly walks away from the carpet but manages to restrain him/herself in time, since this indicates self-control.

| **Questions for meta reflection:** How did you keep in mind that you should not chase after the balloon? What distracting things did we experience today? How could you control your body so that it does not run away after the balloon? Tell me about one thing you did to help your brain to continue to be focused? |
| --- |
| **Closing and transition:** |
| **Vocabulary:** balance, control, focus |
| **Variations and improvements:** Continue to challenge the children by encouraging them to balance the balloon at all sorts of body parts. Pay attention to all children's differences and ensure that they do not become frustrated. The exercise can be used individually, even when other children are sitting down playing. |
| **Possible pitfalls:** The balloons can break, children can collide and children can become frustrated when they fail to balance the balloon. |

**Notes:**
