# Supplementary material for: A randomized controlled trial to examine the effect of two teaching methods on preschool children’s language and communication, executive functions, socioemotional comprehension, and early math skills
Source: BMC Psychol. 2019 Sep 5;7:59. doi: 10.1186/s40359-019-0325-9 (PMC6729003; doi:10.1186/s40359-019-0325-9)
Supplement: Supplementary file 3 — All Univariate results and all Multivariate results. All tests for univariate and multivariate regressions. (PDF 53 kb) [file 40359_2019_325_MOESM3_ESM.pdf]

All univariate results

Outcome variable	Predictor	DF	p	
LANGUAGE_post				
	LANGUAGE_pre	1	<.0001	***
	Intervention	2	0.318	
	Sex	1	0.611	
	SES	1	0.367	
	SECDIwords	1	0.562	
	ECERS	1	0.159	
	SDQ	1	0.478	
	Age	1	0.014	**
	Start	1	0.541	
	BestLanguage	1	0.635	
	L2	1	0.354	
	FLP	1	0.687	
	Fidelity	1	0.269	
(Model DF= 14, error DF= 290, R2= 0.319)				
COMMUNICATION_post				
	COMMUNICATION_pre	1	<.0001	***
	Intervention	2	0.131	
	Sex	1	0.4953	(boys compared to baseline girls)
	SES	1	0.1216	
	SECDIwords	1	0.1139	
	ECERS	1	0.524	
	SDQ	1	0.6088	
	Age	1	0.0854	
	Start	1	0.727	
	BestLanguage	1	0.3817	
	L2	1	0.9388	
	FLP	1	0.0198	**
	Fidelity	1	0.0695	
(Model DF= 14, error DF= 302, R2= 0.371)				
EF_post				
	EF_pre	1	<.0001	***
	Intervention	2	0.179	
	Sex	1	0.762	
	SES	1	0.024	*
	SECDIwords	1	0.886	
	ECERS	1	0.756	
	SDQ	1	0.515	
	Age	1	0.001	***
	Start	1	0.886	
	L2	1	0.878	
	BestLanguage	1	0.737	
	FLP	1	0.152	
	Fidelity	1	0.745	
(Model DF= 14, error DF= 259, R2= 0.636)				
TEC_post				
	TEC_pre	1	<.0001	***
	Intervention	2	0.073	
	Sex	1	0.184	
	SES	1	0.511	
	SECDIwords	1	0.692	
	ECERS	1	0.272	
	SDQ	1	0.242	
	Age	1	0.034	*
	Start	1	0.522	
	BestLanguage	1	0.257	
	L2	1	0.062	
	FLP	1	0.719	
	Fidelity	1	0.014	**
(Model DF= 14, error DF= 326, R2= 0.368)				
MATH_post				
	MATH_pre	1	<.0001	
	Intervention	2	0.892	
	Sex	1	0.211	
	SES	1	0.028	*
	ECERS	1	0.294	
	SDQ	1	0.474	
	Age	1	0.001	***
	Start	1	0.129	
	SECDIwords	1	0.236	
	BestLanguage	1	0.216	
	L2	1	0.233	
	FLP	1	0.605	
	fidelity2	1	0.621	
(Model DF= 14, error DF= 326, R2= 0.565)				

All Multivariate results

Multivariate Analysis of Variance MANOVA Test Criteria and F for the Hypothesis of	Predictor	Wilks' Lambda	F Value	Num DF	Den DF	p
No Overall ECERS*Intervention	ECERS*Intervention	0.947	1.25	10	450	0.258
No Overall SES*Intervention Effect	SES*Intervention	0.935	1.54	10	450	0.124
Interaction effects are nonsignificant, thus fit the multivariate model without these interaction effects below=						
No Overall lang_pre Effect	LANGUGE_pre	0.853	7.89	5	229	<.0001
No Overall comm_pre Effect	COMMUNICATION_pre	0.689	20.63	5	229	<.0001
No OverallEF_pre Effect	EF_pre	0.671	22.49	5	229	<.0001
No OverallTEC_pre Effect	TEC_pre	0.774	13.36	5	229	<.0001
No Overall math_pre Effect	MATH_pre	0.787	12.38	5	229	<.0001
No Overall Intervention Effect	Intervention	0.942	1.38	10	458	0.186
No Overall Sex Effect	Sex	0.991	0.44	5	229	0.824
No Overall fidelity Effect	Fidelity	0.974	1.2	5	229	0.308
No Overall ECERS Effect	ECERS	0.976	1.11	5	229	0.359
No Overall SES Effect	SES	0.963	1.75	5	229	0.123
No Overall FLP (FamilyLanguageProblems) Effect	FLP	0.974	1.23	5	229	0.294
No Overall L2 Effect	L2	0.970	1.4	5	229	0.226
No Overall SDQ Effect	SDQ	0.990	0.45	5	229	0.811
No Overall SECDIwords Effect	SECDIwords	0.995	0.23	5	229	0.949
No Overall Start Effect	Start	0.958	2.03	5	229	0.075
No Overall age Effect	Age	0.962	1.83	5	229	0.108
			Number of Observations Read			432
			Number of Observations Used			251

Estimated effects for the multivariate model

Outcome variables	Parameter	Estimate	SE	t	p	
language_post						
	EF_pre	0.683	0.326	2.1	0.0372	*
	math_pre	-0.010	0.052	-0.19	0.8519	
	language_pre	0.397	0.066	6.05	<.0001	***
	communication_pre	3.635	2.388	1.52	0.1293	
	TEC_pre	0.060	0.140	0.43	0.6664	
communication_post						
	EF_pre	0.001	0.008	0.08	0.9348	
	math_pre	-0.002	0.001	-1.5	0.135	
	language_pre	0.004	0.002	2.56	0.011	*
	communication_pre	0.592	0.059	10.04	<.0001	***
	TEC_pre	0.009	0.003	2.54	0.0116	*
EF_post						
	EF_pre	0.532	0.056	9.54	<.0001	***
	math_pre	0.027	0.009	2.99	0.0031	**
	language_pre	0.015	0.011	1.31	0.1931	
	communication_pre	-0.877	0.409	-2.15	0.033	*
	TEC_pre	-0.002	0.024	-0.07	0.9409	
TEC_post						
	EF_pre	0.389	0.130	3	0.003	**
	math_pre	0.001	0.021	0.03	0.9758	
	language_pre	-0.004	0.026	-0.17	0.8677	
	communication_pre	0.677	0.950	0.71	0.4768	
	TEC_pre	0.440	0.056	7.89	<.0001	***
math_post						
	EF_pre	1.586	0.386	4.1	<.0001	***
	math_pre	0.464	0.061	7.54	<.0001	***
	language_pre	0.002	0.078	0.03	0.9747	
	communication_pre	3.027	2.830	1.07	0.2859	
	TEC_pre	0.004	0.166	0.02	0.9815	
